# Deep Learning to Automate Parameter Extraction and Model Fitting of Two-Dimensional Transistors

**DOI:** 10.34133/research.1103

**Published:** 2026-08-25

**Authors:** Robert K. A. Bennett, Jan-Lucas Uslu, Harmon F. Gault, Asir Intisar Khan, Lauren Hoang, Tara Peña, Kathryn Neilson, Young Suh Song, Zhepeng Zhang, Andrew J. Mannix, Eric Pop

**Affiliations:** ^1^ Department of Electrical Engineering, Stanford University, Stanford, CA 94305, USA.; ^2^ Department of Physics, Stanford University, Stanford, CA 94305, USA.; ^3^ Stanford Institute for Materials and Energy Sciences, SLAC National Accelerator Laboratory, Menlo Park, CA 94025, USA.; ^4^ Department of Materials Science and Engineering, Stanford University, Stanford, CA 94305, USA.; ^5^ Department of Applied Physics, Stanford University, Stanford, CA 94305, USA.

## Abstract

We present a deep learning approach to extract physical parameters (e.g., mobility, Schottky contact barrier height, and defect profiles) of two-dimensional (2D) transistors from electrical measurements, enabling automated parameter extraction and technology computer-aided design (TCAD) fitting. To facilitate this task, we implement a simple data augmentation and pretraining approach by training a secondary neural network to approximate a physics-based device simulator. This method enables high-quality fits after training the neural network on electrical data generated from physics-based simulations of ~500 devices, a factor >40× fewer than other recent efforts. Consequently, fitting can be achieved by training on physically rigorous TCAD models, including complex geometry, self-consistent transport, and electrostatic effects, and is not limited to computationally inexpensive compact models. We apply our approach to reverse-engineer key parameters from experimental monolayer WS_2_ transistors, achieving a median coefficient of determination (*R*^2^) = 0.99 when fitting measured electrical data. We also demonstrate that this approach generalizes and scales well by reverse-engineering electrical data from high-electron-mobility transistors while fitting 35 parameters simultaneously. To facilitate future research on deep learning approaches for the inverse design of transistors, we have published our code and sample datasets online.

## Introduction

Emerging semiconductors, such as two-dimensional (2D) transition metal dichalcogenides (TMDs) [1], amorphous oxides [2], and carbon nanotubes [3], show great promise to enable next-generation electronic devices. Field-effect transistors (FETs) based on these materials are frequently characterized by fitting their measured electrical characteristics to models that express current in terms of either material-level parameters (e.g., semiconductor mobility and Schottky contact barrier height) or device-level parameters (e.g., lumped contact resistances or parasitic capacitances). An advantage of this model fitting approach is that it simultaneously extracts multiple relevant quantities from a few measurements while enabling follow-up simulations to assess performance limitations or circuit-level analyses. However, fitting models to measured data tends to be an iterative process, even for experts in device simulations, making this powerful characterization tool both time-consuming and inaccessible to many researchers [4].

To address these limitations, recent studies have developed neural networks that can predict model parameters from current versus gate-to-source voltage and/or capacitance versus gate-to-source voltage characteristics, achieving excellent fits after training these models on simulated data from 20,000 to 1,000,000 unique devices [5–10]. Acquiring such large training sets is easy for mature technologies with computationally inexpensive models: For example, established compact models for gallium nitride (GaN) [11] or silicon [12] transistors can compute hundreds or thousands of simulated electrical measurements per second. However, emerging FETs are frequently modeled using computationally expensive simulations [13–15]—often because the physics of such devices is more difficult to approximate, or because model development has lagged behind mature technologies—and can require minutes to hours [16,17] to generate a single current versus voltage curve. For this reason, acquiring large datasets for training and fitting emerging device behavior is often intractable.

In this work, we develop a deep learning approach for FET parameter extraction that requires considerably less training data compared to other recent efforts. We reduce the amount of expensive training data by implementing a secondary surrogate network to approximate the electrical model, which we use to generate a large (and computationally inexpensive) augmented dataset that we use to pretrain our primary network. Afterward, we fine-tune the primary network using data generated by the original physics-based model. This approach, from start to finish, requires generating physics-based electrical data from only ~500 devices (>40× fewer compared to previous works) to achieve excellent reverse-engineered fits to experimental data.

We verify our approach using both simulated and experimental test sets, obtaining median coefficients of determination (*R*^2^) fits of 0.995 and 0.990, respectively, when fitting 8 model parameters. We further demonstrate that our approach works for other types of FETs and scales well as the electrical models become more complex (i.e., have more fitting parameters). To facilitate future research, we have also published our datasets and machine learning code (implemented in Python using TensorFlow [18]) in a GitHub repository [19].

## Results and Discussion

### Overview of the deep learning approach

In a standard electrical modeling task, the user inputs a set of model parameters, *Y*_given_ (e.g., carrier mobility and Schottky contact barrier height), into a forward model, ***M***_**forward**_, to compute a current *I*_modeled_:

$$M_{\text{forward}}\left(Y_{\text{given}}\right)=I_{\text{modeled}}$$ (1)

Here, we aim to traina neural network to solve the inverse problem [20,21]. That is, given the measured current *I*_measured_, we aim to train a neural network ***N***_**inverse**_ to predict the set of parameters *Y*_pred_:

$$N_{\text{inverse}}\left(I_{\text{measured}}\right)=Y_{\text{pred}}$$ (2)

such that our original forward model reproduces the measured current:

$$I_{\text{measured}}\approx M_{\text{forward}}\left[N_{\text{inverse}}\left(I_{\text{measured}}\right)\right]=I_{\text{modeled}}$$ (3)

**Fig. 1.**
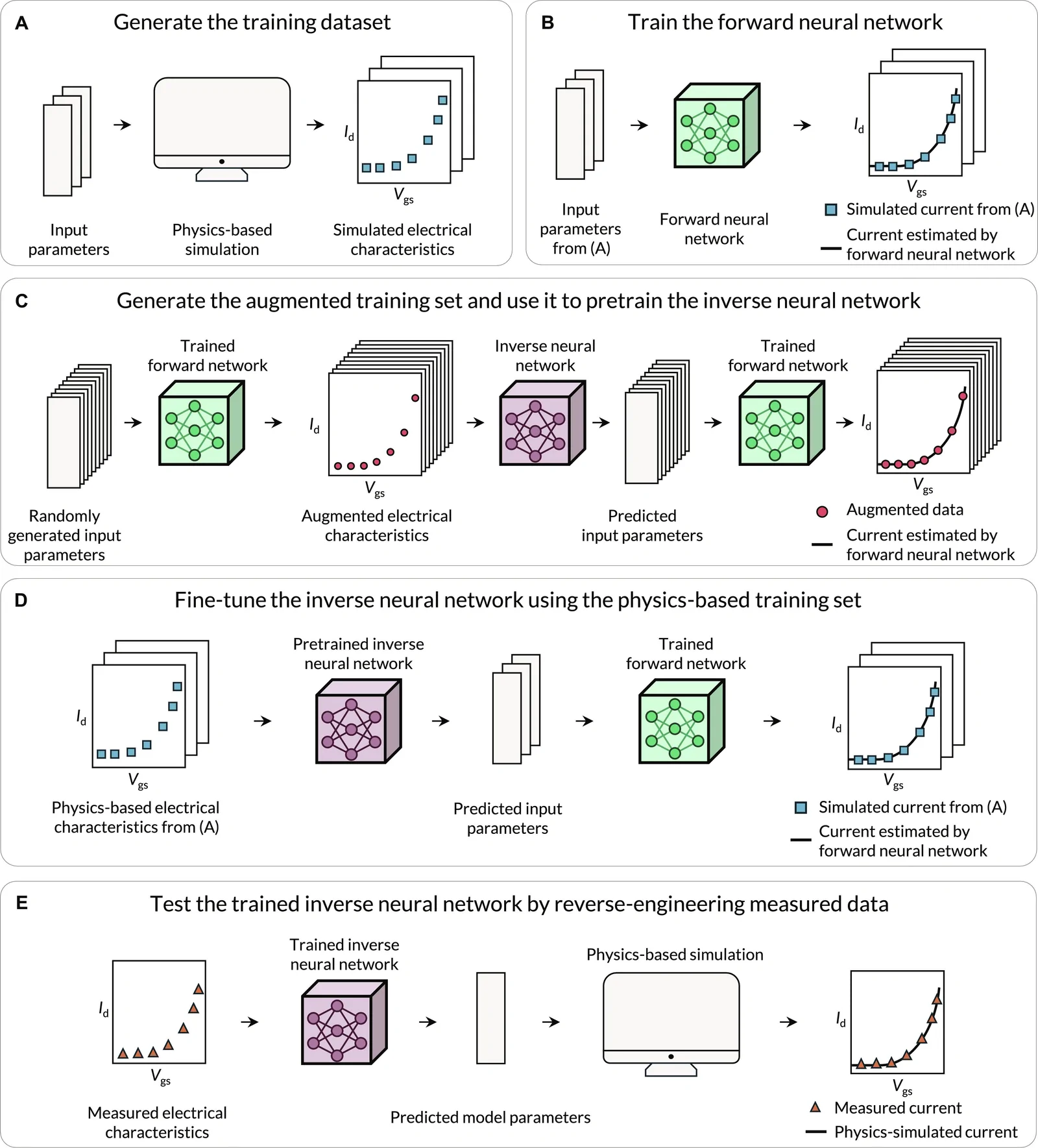
Overview of our deep learning approach for reverse-engineering transistor model parameters. We begin by (A) generating a training set of model input parameters and output electrical characteristics from a physics-based device simulator (here, we use Sentaurus Device [22]). (B) We then train a forward neural network to reproduce the data from the physics-based simulator and (C) use this forward network to generate an augmented dataset with which we pretrain the inverse neural network, and then we finish training on the original physics-simulated electrical data (D). In (C) and (D), we train the inverse neural network to predict model parameters that will reproduce the original input current; we estimate the current during training by calling the trained forward neural network from step (B) to do so (i.e., as a tandem neural network). (E) Next, we test the trained inverse neural network by providing it with new electrical data to be reverse-engineered, extracting the model parameters, and running new physics-based simulations using the predicted parameters. Finally, we plot the new simulated curves alongside the original measured data to assess the quality of the fit.

Our approach for accomplishing this task is summarized in Fig. 1 and below:

1. Use a comprehensive physics-based model (e.g., Sentaurus Device [22]) to generate a training dataset of input parameters and calculated electrical characteristics (Fig. 1A).
2. Train an intermediate forward neural network to approximate the physics-based model (Fig. 1B).
3. Generate a much larger augmented training set using the forward neural network and use this augmented data to pretrain the inverse neural network, calling the forward neural network repeatedly to guide training as a tandem neural network (Fig. 1C) [23].
4. Finish training the inverse neural network using the original dataset from step 1, once again calling the forward neural network repeatedly to guide training as a tandem neural network (Fig. 1D).
5. Test the inverse neural network by inputting new electrical data, extracting the model parameters, and calling the physics-based model on the predicted model parameters to assess the accuracy of the extraction (Fig. 1E).

### Generating the training set with a physics-based simulator

We generate our training data using Sentaurus Device [22], an industry-standard technology computer-aided design (TCAD) program, as our physics-based model. This simulator could be any software that generates complete current versus gate-to-source voltage data based on physical input parameters such as device geometry and material properties; other examples include Monte Carlo [24] or quantum transport simulators [14,15].

For any fit to provide valid physical insights, regardless of how the fit is achieved—through conventional techniques or otherwise—the user must take care to ensure that the model used to generate the training data accurately describes their transistors. For instance, with enough fitting parameters, many models could enable a close fit between measured and modeled data, even if the physics of the model are wrong. However, for some applications, this could also be acceptable: For instance, empirical “phenomenological” models [25] and surrogate models [26] can be practical for circuit-level SPICE [27] simulations. In this work, we aim to provide an automated method to achieve a fit to a model that the user specifies, rather than to automate the process of selecting this model. For this fit to provide meaningful physical insight, it is therefore essential that the user select a physically meaningful model.

**Fig. 2.**
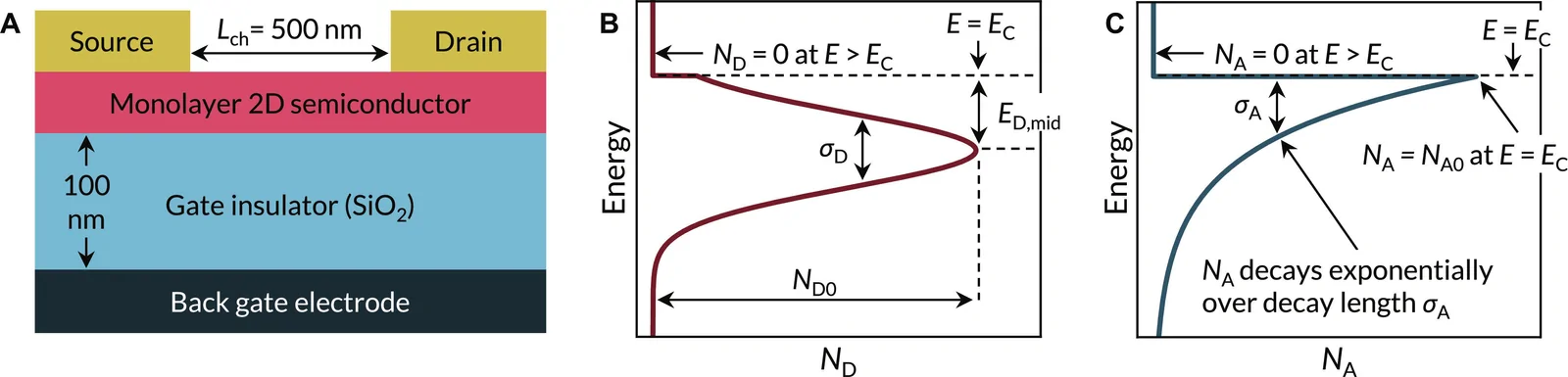
Summary of transistors considered in this work. (A) Diagram of our transistor geometry, showing a 2D semiconductor channel integrated in a back-gated transistor (not to scale). (B) Donor-like defect profile *N*_D_ and (C) acceptor-like band-tail *N*_A_. These profiles are given in Eqs. (4) and (5), respectively, and are parameterized in terms of the model parameters listed in Table 1.

In our work, we use Sentaurus Device to generate *I*_d_–*V*_gs_ curves (where *I*_d_ is the drain current and *V*_gs_ is the gate-to-source voltage) for 2D FETs using the device geometry shown in Fig. 2A. This back-gated geometry is frequently used in experimental studies on 2D or other emerging semiconductors because it is easier to fabricate than top- or dual-gated devices; we use this structure here to allow us to easily test our neural networks on readily available experimental *I*_d_–*V*_gs_ data from monolayer semiconductor transistors. We include additional Sentaurus Device simulation details in Section S1. We discuss how this approach may be generalized to other types of transistors later in this work.

To build our training set, we initialize *n*-type FETs in Sentaurus Device TCAD [22] with the geometry shown in Fig. 2A, choosing the value of each model parameter at random from the ranges listed in Table 1. Here, we intentionally use large ranges for each parameter to ensure that the neural network can fit a wide range of device data, as the neural network cannot predict the values of parameters outside of these ranges. We emphasize that the 8 parameters in Table 1 are the fitting parameters considered in this work. Other semiconductor-level parameters (e.g., band gap and dielectric permittivity) and device-level parameters (e.g., channel length and oxide thickness) are treated as fixed model inputs for all simulations, as described in Section S1.

**Table 1.** Model parameters and the ranges across which they vary in the physics-based training set. *N*_D0_, *E*_D,mid_, and *σ*_D_ characterize donor-like defects, and *N*_A0_ and *σ*_A_ characterize acceptor-like band-tail states. The defect-like and acceptor-like profiles are shown in Fig. 2B and C, respectively. We elaborate upon the physical meaning of these parameters and justify these selected ranges based on experimental and theoretical considerations in Section S2.

Parameter	Range	Unit
Mobility *μ*	1–35	cm^2^ V^−1^ s^−1^
Schottky contact barrier height *ϕ*_B_	10–510	meV
Effective density of states *N*_C_	2 × 10^11^–9 × 10^12^	cm^−2^
Peak donor density *N*_D0_	3 × 10^12^–3 × 10^13^	eV^−1^ cm^−2^
Donor energy mid *E*_D,mid_	20–200	meV below *E*_C_
Donor energy width *σ*_D_	20–200	meV
Peak acceptor band tail density *N*_A0_	6 × 10^11^–3.7 × 10^13^	eV^−1^ cm^−2^
Acceptor band tail energy width *σ*_A_	50–300	meV

With this approach, we then simulate sets of *I*_d_–*V*_gs_ characteristics for 25,000 devices from *V*_gs_ = −6 to 50 V at *V*_ds_ = 0.1 and 1.0 V, creating a large set of established *I*_d_–*V*_gs_ versus model input parameter relationships with which we train our neural networks. We will later use subsets of this larger training set in bootstrap studies to determine the minimum acceptable training set size.

We note that if the distribution of any individual parameter is narrower than listed here, then the ranges of these parameters should be adapted accordingly. In general, reducing the allowable range of fitting parameters will make the fitting task easier; however, doing so requires one to be confident in the range of values a given parameter may take, which may not always be possible, especially for emerging semiconductor devices with unoptimized fabrication protocols.

It is also essential to note that the data used to train the neural networks must reflect the technology that the networks will be applied to. For instance, to build a neural network that will be used for the inverse design of another 2D semiconductor, the TCAD model used to build the training set should be updated to reflect the specifics of that semiconductor, and the parameters in Table 1 should be adjusted to ensure that they lie within their expected values for the experimental devices of interest.

### Model parameters for 2D transistors

The parameters we aim to fit include the electron mobility *μ*, Schottky contact barrier height *ϕ*_B_, and effective density of states at the conduction band edge *N*_C_. These parameters capture, respectively, the speed at which electrons can move through a semiconductor, the energy barrier associated with electron injection between the semiconductor and metal, and the number of available states for electrons in the semiconductor. The remaining parameters quantify defect profiles in 2D devices. Donor states (e.g., chalcogen vacancies; see Section S2) follow a truncated Gaussian distribution (Fig. 2B) with respect to energy *E*,

$$\begin{array}{c} N_{D}\left(E\right)=N_{D0}\;\text{exp}\left(-\frac{{\left(E-E_{D,\mathrm{mid}}\right)}^{2}}{2\sigma_{D}^{2}}\right)\;\text{for}\;E<E_{C}\\ N_{D}\left(E\right)=0\;\text{otherwise} \end{array}$$ (4)

where *N*_D0_ is the peak donor density, *E*_D,mid_ is the energy at which the Gaussian donor distribution peaks (referenced to the conduction band edge *E*_C_), and *σ*_D_ is the standard deviation (width) of the Gaussian curve.

We also include trap-like acceptor states (Fig. 2C), which could arise from dispersionless band-tail states (see Section S2). Their distribution decays exponentially from the conduction band edge *E*_C_:

$$\begin{array}{c} N_{A}\left(E\right)=N_{A0}\;\text{exp}\left(-\left|\frac{E-E_{C}}{\sigma_{A}}\right|\right)\;\text{for}\;E<E_{C}\\ N_{A}\left(E\right)=0\;\text{otherwise} \end{array}$$ (5)

where *N*_A0_ is the peak acceptor density and *σ*_A_ is the characteristic decay energy. We elaborate upon the physical meaning of each fitting parameter in Section S2.

In this work, we use only synthetic (i.e., TCAD and compact model) data to train our neural networks. In principle, experimental data could also be incorporated into these training sets, although it may be difficult to obtain a sufficiently large labeled dataset with purely experimental data, particularly for 2D semiconductors today. To incorporate experimental data when training a similar model, one could therefore use a “hybrid” dataset, i.e., one with both experimental and TCAD data, while oversampling the experimental data [28].

### Training the inverse neural network in tandem with a forward solver

**Fig. 3.**
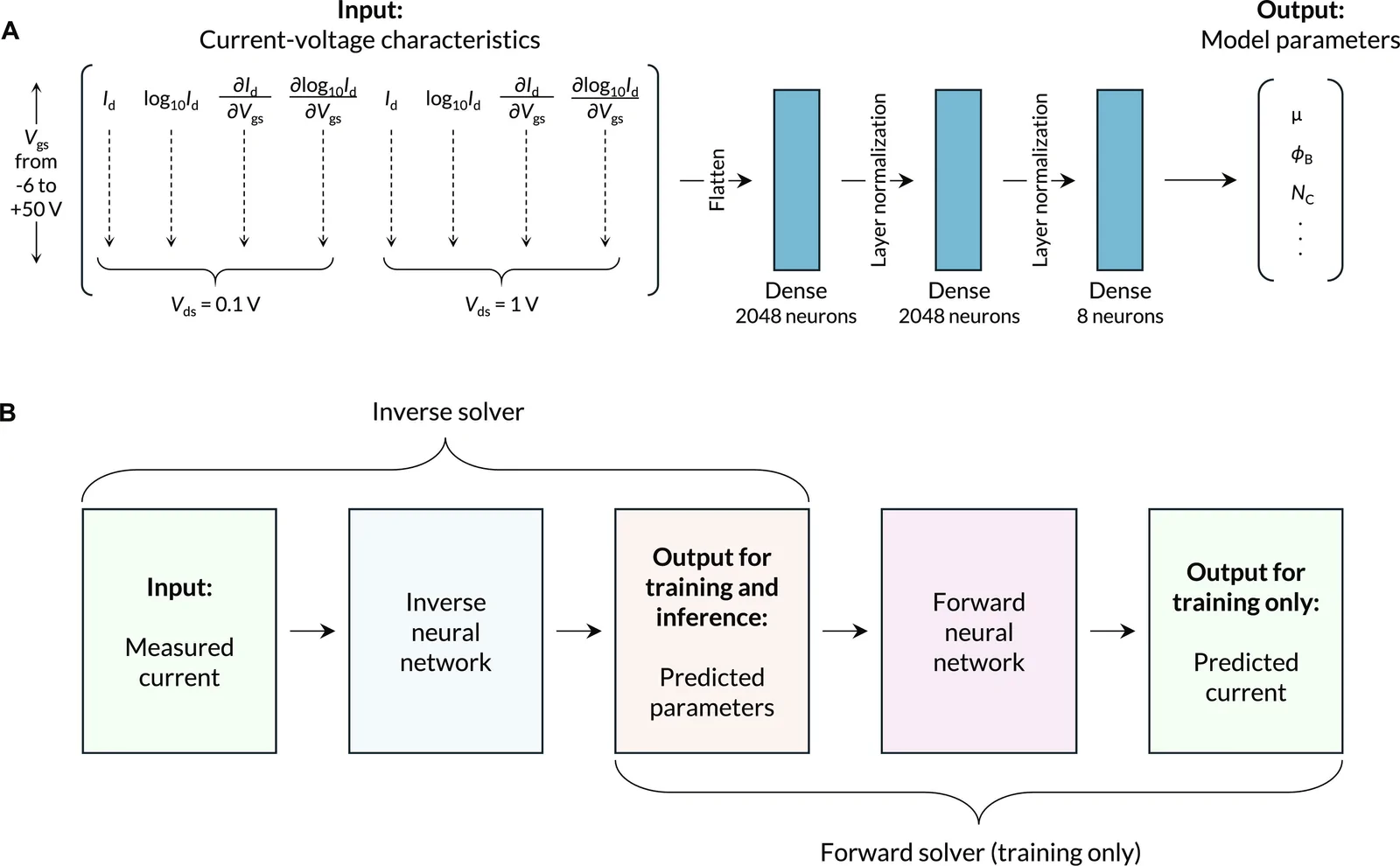
Inverse neural network and tandem network training approach. (A) Architecture of the inverse neural network. Each dense layer uses the rectified linear unit (ReLU) activation function, except the final layer, which uses the hyperbolic tangent (tanh) activation function. The network accepts current versus gate-to-source voltage (*I*_d_–*V*_gs_) measurements in linear and logarithmic space, along with their derivatives with respect to *V*_gs_, and outputs the set of model parameters that a physics-based solver can use to reproduce these input data. (B) Tandem approach used for training the inverse neural network. During training, the output of the inverse neural network is fed into a pretrained forward neural network, which allows us to include errors of predicted current in the loss function. We use this tandem approach only while training; during inference, the network outputs the predicted parameters directly. Details of the training procedures and descriptions of the forward neural network are given in Materials and Methods, and in Sections S4 and S5.

The inverse neural network architecture used in this work is shown in Fig. 3A. This neural network accepts *I*_d_–*V*_gs_ data from physics-based simulations (*V*_ds_ = 0.1 and 1.0 V, with 32 *V*_gs_ values evenly spaced from −6 to +50 V); we also perform simple feature engineering by explicitly inputting ∂*I*_d_/∂*V*_gs_, log_10_*I*_d_, and ∂log_10_*I*_d_/∂*V*_gs_ for each *V*_ds_, yielding 8 vectors that we input into the neural network as a 32 × 8 matrix. We discuss and justify this feature engineering in Section S3. Other recent works on machine learning-based parameter extraction have also included capacitance–voltage data as input features for silicon-based devices [9]. Although including such data would likely facilitate training and fitting, these capacitances are not consistently measured for 2D devices today, unlike *I*_d_–*V*_gs_ characteristics. To ensure that our approach remains applicable to experimental 2D transistors without demanding additional measurements, we avoid relying upon these capacitance measurements here. Furthermore, while extremely low currents (e.g., <1 pA/μm) are readily simulated, they remain difficult to measure experimentally due to noise floors imposed by hardware. We therefore intentionally impose an artificial noise floor on all training data, as discussed in Section S4, to avoid training our neural networks on these difficult-to-measure data.

The inverse neural network aims to output the set of Sentaurus model parameters associated with these *I*_d_–*V*_gs_ curves and derivatives. To accomplish this, we use the tandem neural network approach summarized in Fig. 3B. Such tandem neural networks have found widespread use in many inverse design tasks [23,29,30], including transistor parameter extraction [7]. In this approach, the inverse neural network is trained by feeding its output (i.e., the Sentaurus Device model parameters) into a pretrained forward neural network, which then outputs the estimated *I*_d_–*V*_gs_ characteristics corresponding to those model parameters. In other words, the forward neural network approximates the original physics-based TCAD solver or, equivalently, it approximates ***M***_**forward**_ in Eq. (1). We describe all scaling, preprocessing, and training procedures in Materials and Methods (Neural network training details) and in Section S4. We describe the architecture of the forward neural network and characterize its performance in detail in Section S5.

A tandem approach is necessary here because *I*_d_–*V*_gs_ characteristics are generally not unique functions of the model parameters [7]. In other words, there are often multiple sets of input parameters that can allow the physics-based simulator to yield identical or near-identical *I*_d_ profiles, making it difficult to train the inverse neural network based on errors in parameters alone. Instead, training the inverse neural network using a tandem approach allows us to incorporate errors in both parameters and predicted current into the loss function,

$$L=\sqrt{\frac{1}{N_{\text{devices}}N_{\text{params}}}\sum_{i}^{N_{\text{devices}}}\sum_{l}^{N_{\text{params}}}{\left(Y_{\text{actual}}^{\left(i,l\right)}-Y_{\text{pred}}^{\left(i,l\right)}\right)}^{2}\cdot \frac{1}{N_{\text{devices}}}\sum_{i}^{N_{\text{devices}}}E_{I_{d}}^{\left(i\right)}}$$ (6)

where *N*_devices_ is the number of devices, *N*_params_ is the number of fitting parameters in the parameter set *Y*, the subscripts “actual” and “pred” denote baseline truths and predicted quantities, and the superscripts (*i*) and (*ℓ*) denote the *i*th device and *ℓ*th fitting parameter, respectively. $E_{I_{d}}^{\left(i\right)}$ is the error in *I*_d_ between the actual and predicted currents and their derivatives, given by Eq. (S1) in Section S4. During training, we estimate the predicted current $I_{\text{pred}}^{\left(i\right)}\;$by calling the forward network on the predicted parameter set, i.e., $I_{\text{pred}}^{\left(i\right)}=M_{\text{forward}}\left(Y_{\text{pred}}^{\left(i\right)}\right)$.

We emphasize that we use this tandem approach only when training the inverse neural network, not for inference.

### Pretraining with an augmented dataset and fine-tuning with physics-based data

We take advantage of the forward neural network to build an augmented dataset that we use to pretrain our inverse network. Here, we generate many sets of randomly chosen input parameters using the same parameters and ranges from Table 1 and then use the forward network to estimate the corresponding *I*_d_–*V*_gs_ curves. These augmented data are essentially free compared to our original physics-based simulated data: It takes ~1 min to generate a single *I*_d_–*V*_gs_ curve with Sentaurus Device (for other device simulators, this time can vary greatly, depending on the specific device and models used), whereas we can generate 100,000 *I*_d_–*V*_gs_ curves with the forward neural network in less than 30 s. After pretraining our inverse neural network on this augmented dataset, we use the physics-based simulation training set to fine-tune the model. We detail full training procedures in Section S4.

After training, we test the performance of the inverse neural network by using it to extract estimated model parameters from a Sentaurus Device-simulated test set of 1,000 devices (generated with the same procedure as training and development sets) that was unseen by the neural network during training and network optimization. Afterward, we take these extracted parameters and estimate their corresponding *I*_d_–*V*_gs_ curves using a well-trained forward neural network that was trained on all 25,000 devices in our original physics-based training set. We show in Section S5 that this forward neural network reproduces our original Sentaurus simulations with exceptional accuracy; thus, this approach allows us to estimate the accuracy of the inverse neural network without having to re-run Sentaurus Device simulations, which can become computationally expensive when repeatedly evaluating the performance of the inverse neural network on multiple training sizes. Finally, we calculate *R*^2^ between the original and newly obtained *I*_d_–*V*_gs_ characteristics to quantify how accurately these estimated parameters reproduce the original *I*_d_–*V*_gs_ curves (higher *R*^2^ is better; *R*^2^ = 1 is a perfect fit; details of the *R*^2^ calculation are in Section S4).

Our original physics-based training set contains *I*_d_–*V*_gs_ curves from 25,000 unique devices; however, we wish to develop a machine learning approach that relies upon as few simulations as possible, since they are computationally expensive. Thus, we apply the bootstrap approach described in Section S4 to draw smaller training sets to quantify how the training set size impacts the performance of the neural network. Note that when we refer to the training set size, we refer to the number of device configurations used for physics-based simulations in both the training and development sets combined and that for each bootstrapped training procedure, we use the same subset of data to train both the forward and inverse networks. For example, if we specify that the training set size is 500, we (a) use 500 sets of physics-based simulations to train the forward neural network, (b) use that forward neural network to generate a large augmented dataset (100,000 sets of *I*_d_–*V*_gs_ curves) with which we train the inverse neural network, and then (c) fine-tune the inverse neural network on the original 500 physics-based simulations.

**Fig. 4.**
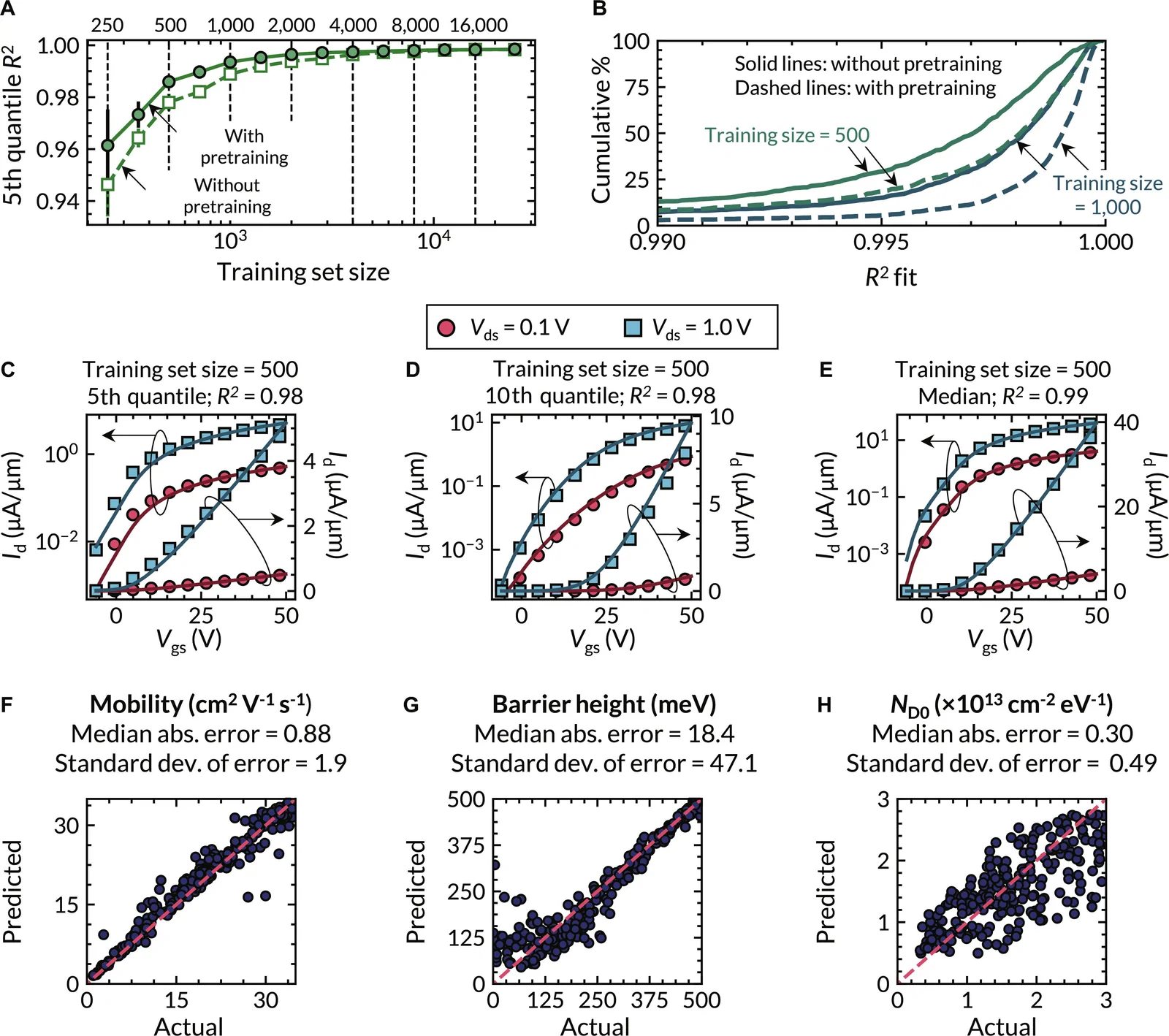
Performance of the inverse network on the simulated test set. (A) Fifth quantile (i.e., worst 5%) *R*^2^ fits attained by the inverse neural network as a function of training size, with and without pretraining, and (B) sample cumulative *R*^2^ fits achieved by the inverse neural network, with (solid lines) and without (dashed lines) pretraining. Error bars in (A) show standard deviations across 5 bootstrapped runs; error bars are often small and are not always visible. Sample current versus gate-to-source voltage fits after training the inverse neural network on 500 devices, showing the reverse-engineered fits corresponding to (C) the 5th quantile, (D) 10th quantile, and (E) median *R*^2^ (symbols: original physics-based simulations; lines: estimated *I*_d_–*V*_gs_ curves obtained using reverse-engineered parameters). The data are shown on both logarithmic (left) and linear axes (right), at *V*_ds_ = 0.1 V (red) and 1.0 V (blue). Actual versus predicted values for (F) mobility, (G) Schottky contact barrier height, and (H) peak donor density. The pink dashed lines are the lines of zero error, i.e., where predicted and actual values match perfectly.

We plot the fifth quantile (worst 5%) *R*^2^ for various training set sizes, with and without pretraining, in Fig. 4A, and show sample histograms for training sets consisting of 500 and 1,000 devices in Fig. 4B. Here, we find that pretraining greatly improves the performance of the inverse neural network; in terms of *R*^2^ fit, implementing this pretraining procedure appears to offer an accuracy boost roughly equivalent to doubling the size of the original physics-based training set. We show sample fits corresponding to the 5th quantile (worst 5%), 10th quantile (worst 10%), and median *R*^2^ in Fig. 4C to E for an inverse neural network with pretraining (500 devices in the physics-based training set), confirming a good match between the original simulated *I*_d_–*V*_gs_ data and that obtained with the reverse-engineered parameters.

Our approach sharply reduces the number of physics-based (Sentaurus Device TCAD) simulations needed to achieve excellent fits across the majority of the test set compared to previous studies. These past efforts have used training sets consisting of 20,000 to 1,000,000 devices to achieve high-quality fits for a similar reverse-engineering task, whereas we demonstrate excellent fits using training sets of ~500 devices, reducing the required number of training devices by ~40×. For this training set size, the full training procedure takes less than 5 min (including training the forward network, generating the augmented dataset, and then pretraining and fine-tuning the inverse neural network) on an NVIDIA RTX 4080 graphics processing unit (GPU), which is much less time compared to that required to generate the physics-based data.

The pretraining approach we implement is especially beneficial when fitting data to computationally expensive TCAD models, since we reduce the number of time-consuming simulations required to build the training set. This pretraining approach is less useful when fitting computationally inexpensive models (e.g., compact models implemented in Verilog-A), where large datasets are easily and cheaply obtained. However, even when simulated *I*_d_–*V*_gs_ data are inexpensive, we emphasize that the surrogate model still facilitates training by providing a GPU-compatible means to evaluate the $E_{I_{d}}^{\left(i\right)}$ term in the loss function [Eq. (6)]. Therefore, training a surrogate model (as in Fig. 1B) remains advisable for this inverse design approach, even when the target model is computationally inexpensive.

This reduction in training set size makes it feasible to train the neural network on data generated by computationally intensive models: For example, assuming parallelization to 32 cores, it would take ~30 min to generate a 500-device training set if using a TCAD solver like Sentaurus with ~2 min per *I*_d_–*V*_gs_ curve, or ~1.25 d if using a quantum transport solver (e.g., the non-equilibrium Green’s function method) with ~1 h per *I*_d_–*V*_gs_ curve. It would take ~1 d or ~1 month (respectively) to generate these training sets if using a fitting approach that requires training on 20,000 devices; thus, the approach presented here reduces a day-long generation task to something that can be accomplished in under an hour, or an (often) unfeasibly long task to something that can be accomplished in a day.

Next, we investigate the accuracy of the extracted parameters, plotting the actual versus predicted values for mobility, Schottky contact barrier height, and peak donor density in Fig. 4F to H. We show similar plots for all model parameters in Section S6. Here, we find that the mobility and Schottky barrier height can both be extracted with relatively low median absolute errors of 0.88 cm^2^ V^−1^ s^−1^ and 18.4 meV, respectively, representing 2.6% and 3.7% of their nominal ranges. The standard deviation of the mobility error is also quite small at 1.9 cm^2^ V^−1^ s^−1^ (5.6% of its nominal range), whereas the standard deviation for the Schottky barrier height (47.1 meV) is 9.4% of its nominal range. However, from Fig. 4G, most of these errors occur when the actual Schottky barrier height is comparable to the thermal energy *k*_B_*T* (where *k*_B_ is the Boltzmann constant and *T* = 300 K is the absolute temperature), which makes sense physically: Once the barrier is “small enough,” small deviations will not meaningfully affect the contact resistance or the resultant *I*_d_.

We find larger errors in the other model parameters—for example, as shown in Fig. 4H, the median absolute error for the peak donor density *N*_D0_ (3.0 × 10^12^ cm^−2^ eV^−1^) represents 11.1% of its nominal range, and the relatively large standard deviation (4.9 × 10^12^ cm^−2^ eV^−1^) represents 18.1% of this range. We speculate that we see larger errors in these parameters because they have some mutual redundancy: For example, if the peak acceptor density is underestimated, then the overall acceptor profile could still be approximated by increasing its energy width to compensate, as we demonstrate in Section S6. In comparison, the mobility and Schottky contact barrier height both influence the *I*_d_–*V*_gs_ curves uniquely compared to other parameters. In other words, if these parameters are estimated incorrectly, the overall *I*_d_–*V*_gs_ curves cannot be matched by adjusting other model parameters to compensate, leading to lower error in their extractions.

We note that for some applications, like circuit-level modeling, large errors in individual parameters may be acceptable as long as the fitted model can accurately reproduce experimental *I*_d_–*V*_gs_ (and/or *I*_d_–*V*_ds_) data. Alternatively, for applications where strong physical insights are needed—such as monitoring the quality of a growth by estimating the defect density in the semiconductor—the accuracy of individual parameters for a specific device may be essential. We discuss strategies for estimating the uncertainty for these individual parameters in Section S6.

### Experimental validation using measured *I*_d_–*V*_gs_ data from monolayer WS_2_ transistors

**Fig. 5.**
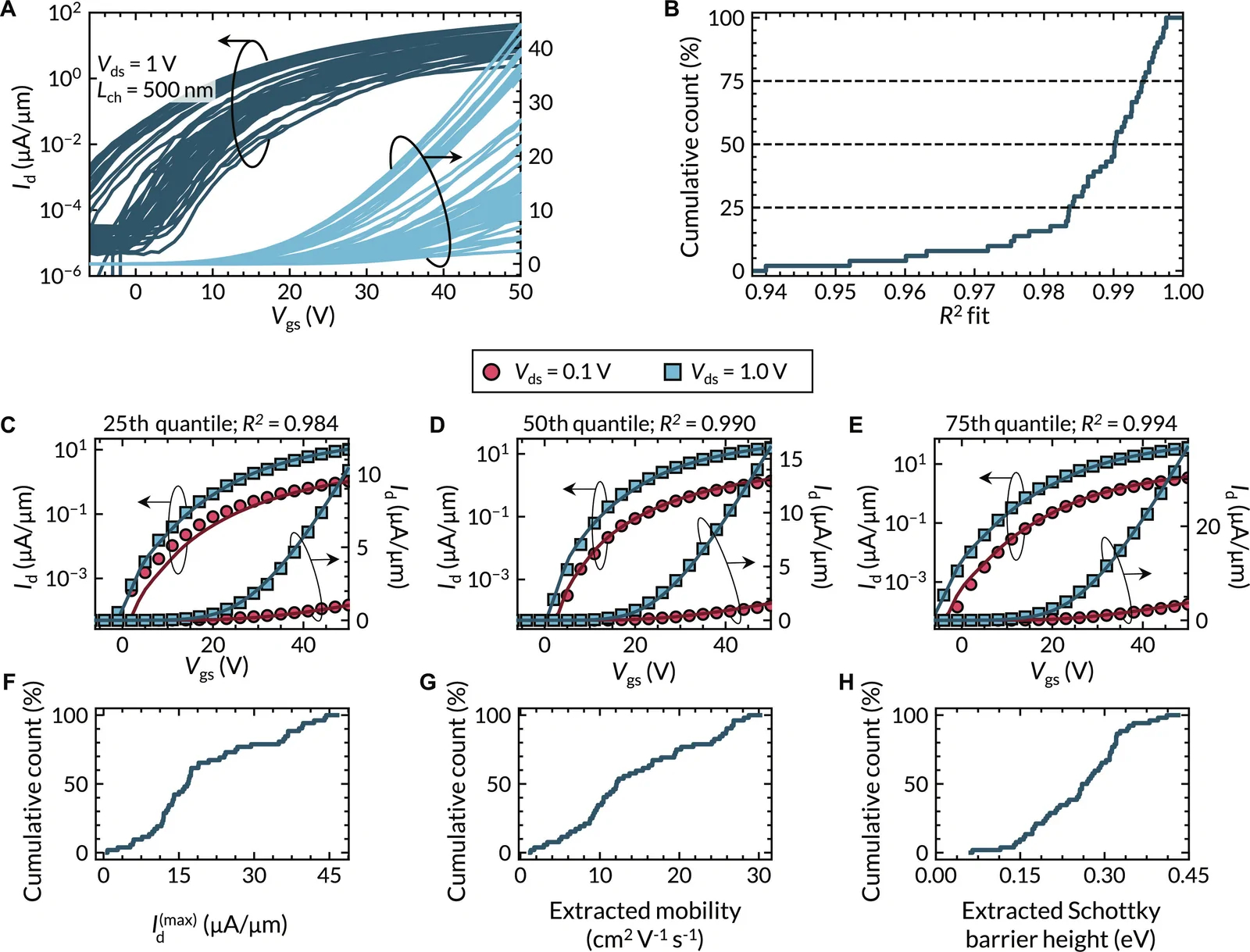
Performance of the inverse network on experimentally measured test devices. (A) All 51 measured *I*_d_–*V*_gs_ curves for monolayer WS_2_ transistors (*V*_ds_ = 1 V) in our experimental test set, shown both on logarithmic (left) and linear (right) *y* axes. (B) Cumulative histogram showing the *R*^2^ fits between the original experimental data and devices reverse-engineered by an inverse neural network trained on 500 sets of physics-based simulations. Sample *I*_d_–*V*_gs_ fits for (C) the 25th quantile, (D) median, and (E) 75th quantile *R*^2^, showing good agreement between the experimental data (symbols) and our reverse-engineered fits (lines). Data are shown on both logarithmic (left) and linear axes (right), at *V*_ds_ = 0.1 V (red) and 1.0 V (blue). Cumulative histograms for (F) the experimental *I*_d_^(max)^, (G) the extracted mobility, and (H) the extracted Schottky barrier height.

We validate our deep learning approach experimentally by using a test set collected from 52 monolayer WS_2_ transistors that were fabricated across 3 separate chips [31]. All measured working devices were included, except for one outlier that could not achieve an on-state current of at least 1 μA/μm at *V*_gs_ = 50 V and *V*_ds_ = 1 V, leaving a total of 51 devices in the test set. These devices follow the geometry shown in Fig. 2A with a channel length of 500 nm, 100 nm SiO_2_ back-gate insulators, and nickel contacts (full experimental details are described in Materials and Methods under Experimental details for monolayer WS_2_ transistors). We choose this channel length because it offers an intermediate regime where both the channel and contacts will influence *I*_d_, allowing us to characterize both the channel and contacts. To match our physics-based training sets, we perform *I*_d_–*V*_gs_ sweeps at *V*_ds_ = 0.1 and 1 V (all curves at *V*_ds_ = 1 V are plotted in Fig. 5A) and then feed the measured data into our inverse network after training it on physics-based simulations from 500 devices. We then extract the 8 model parameters listed in Table 1 and perform new Sentaurus TCAD simulations using these predicted parameters.

We plot the cumulative *R*^2^ between the TCAD-simulated *I*_d_–*V*_gs_ curves (simulated using the predicted input parameters) and original experimental data in Fig. 5B, finding that the 25th quantile (worst 25%), median, and 75th quantile (best 25%) *R*^2^ fits for the test sets are 0.984 (95% confidence interval: 0.979 to 0.987), 0.990 (95% confidence interval: 0.986 to 0.993), and 0.994 (95% confidence interval: 0.992 to 0.996), respectively, where confidence intervals are estimated by the empirical bootstrap method [32]. We plot the corresponding *I*_d_–*V*_gs_ fits in Fig. 5C to E, along with fits for all 51 experimental devices in Section S7.

A key advantage of this deep learning approach is that once the neural networks are trained, we can rapidly characterize many devices simultaneously to study variation across one or more chips. For example, we plot the cumulative histogram of the maximum measured current at *V*_gs_ = 50 V and *V*_ds_ = 1 V, *I*_d_^(max)^, in Fig. 5F, as well as the extracted electron mobility and Schottky contact barrier heights in Fig. 5G and H. We find that the extracted mobility and barrier heights show considerable variation, agreeing well (qualitatively) with the experimental variation in Fig. 5A and F. Overall, we anticipate that this approach could be used to facilitate characterization and analysis of large distributions of devices, so long as the ranges of variables in the training set are sufficiently broad to encapsulate the variation of these relevant parameters experimentally.

Figure 5G and H also address questions about the distribution of electron mobility and contact resistance one can expect for a particular WS_2_ synthesis and fabrication approach. For example, the mobility includes information about the electron effective mass and scattering mechanisms with lattice vibrations and defects, both in the WS_2_ film and its substrate. In other words, Fig. 5G could be used to obtain information about the defect distribution in such WS_2_ films. Similarly, Fig. 5H can be used to understand how the deposition of the metal (nickel) affects the transistor contacts. We caution that the distributions shown here are for unoptimized academic devices. One must increase the mobility, decrease the Schottky barrier height, and tighten these distributions before these transistors can become competitive in the semiconductor industry.

We emphasize that in model fitting, a close match between the original data and the model fit (i.e., a fit that yields *R*^2^ ≈ 1) does not guarantee that the extracted parameters are necessarily accurate. For example, a high-quality fit could also be achieved by the model overfitting the data. This is a well-known limitation of model fitting that must be considered when interpreting the results of any parameter extraction (whether the fit is achieved manually, through conventional techniques, or using the deep learning method in this work). To partially address issues arising from overfitting, one could also incorporate temperature-dependent data, incorporate hysteresis explicitly, add additional *I*_d_–*V*_gs_ curves (at additional fixed *V*_ds_ values), and/or add *I*_d_–*V*_ds_ curves (at multiple fixed *V*_gs_ points), and enable electrical models computed self-consistently with device heating effects. These approaches could reduce the number of parameter sets that yield near-identical fits across the entire range of data.

### Model generalization and fitting many physical parameters simultaneously

Until now, we have confined our discussion to 2D semiconductor transistors simulated by TCAD using 8 fitting parameters. However, model fitting is routinely applied to other types of devices, often using highly dimensionalized models with many parameters [11,12]. In Section S8, we study how our approach scales with the complexity of the *I*_d_–*V*_gs_ model by applying the same methodology to train a neural network to predict the input parameters of GaN high-electron-mobility transistors (HEMTs) simulated with the industry-standard Advanced Spice Model for HEMTs (ASM-HEMT) compact model [11]. We find that training becomes more difficult when fitting more physical transistor parameters simultaneously; this result is an expected consequence of the curse of dimensionality [33] encountered when fitting high-dimensional data, as the size of the parameter space grows exponentially as additional parameters are added (e.g., a space with *N* dimensions, each sampled along *k* values, has size *k^N^*). Nevertheless, we demonstrate that we can still achieve good fits when fitting 15 or 25 or 35 parameters with a training set containing data from ~1,000 or 4,000 or 16,000 unique devices, respectively.

These scaling trends indicate that the method proposed in this work could be applied to fit transistor data even to complicated models with many unknown parameters, as long as generating the appropriately sized training set remains computationally tractable. This consideration varies tremendously from model to model: For example, generating such large training sets could be trivial for compact models implemented in Verilog-A, including the ASM-HEMT model [11] considered above, which can be used to generate thousands of *I*_d_–*V*_gs_ curves per second. It may be considerably harder to generate such large datasets with rigorous physics-based models, especially in the face of hardware limitations and/or limited licenses, which may restrict parallelizability.

Once a neural network is fully trained, it can be used repeatedly to fit many devices with minimal computation cost, as we do with experimental devices in Fig. 5. In other words, the process of acquiring training data is a large “up front” cost; however, once the model is fully trained, model inference (i.e., applying the inverse neural network to extract device parameters from *I*_d_–*V*_gs_ curves) takes only a small fraction of 1 s. Therefore, this deep learning approach is especially appealing when a model fitting procedure must be repeated multiple times, e.g., to monitor the performance of a device over time, to quantify the performance of devices on a production line, or to fit many devices in a large experimental dataset simultaneously. Alternatively, if only a single device needs to be fit to a TCAD or compact model, then another fitting approach, e.g., manual fitting or derivative-free optimization [4], could incur a lower total computational cost compared to the deep learning approach studied here.

## Conclusion

In conclusion, we have developed a deep learning approach to automate parameter extraction (TCAD fitting) for transistors based on their current versus gate-to-source voltage characteristics. We greatly reduce the number of devices required in our training set compared to those used in prior works, and we demonstrate excellent fits on experimental 2D transistors using models trained on physics-based simulations from 500 devices. This approach also generalizes well to other types of transistors and models (e.g., GaN HEMTs with up to 35 unknown parameters in the ASM-HEMT model), as long as acquiring larger training sets remains computationally feasible. Thus, we anticipate that the results of this study will facilitate fitting experimental device measurements to both physics-based and compact models alike. Furthermore, machine learning models have recently been shown to be efficient for modeling emerging technologies, including static random access memory (SRAM) [34], 3D NAND memory [35], and 2D terahertz transistors [36]. Therefore, the inverse design approach we present here could facilitate the design and analysis of these and other devices.

In this work, we have not investigated how noisy or partially corrupted measurements affect the accuracy of this method. To make this fitting method more robust against noise and corruption, these factors could be incorporated into the training data, e.g., by imposing artificial noise or by artificially corrupting data within the datasets used to train the neural networks. Further, engineering the loss function, e.g., as implemented in a previous work [4], could offer another promising method of making this machine learning approach more robust.

A disadvantage of this machine learning framework (and similar approaches put forward in previous works [5–10]) is that fitting devices with different geometries (e.g., varying channel length or oxide thickness, or changing the gating configuration between bottom-, top-, or dual-gated devices) requires that training sets be regenerated and neural networks be retrained. To address this issue, one could explicitly include channel length and other relevant geometry parameters as flexible inputs when training the forward and inverse neural networks. Alternatively, neural networks could be adapted across geometries via transfer learning [37], i.e., using a tandem network originally pretrained on one geometry, and then using less fine-tuning data to adapt it to a different device geometry. Both approaches are promising research directions for future work that would boost the robustness and practicality of the deep learning method implemented here.

To facilitate these future research directions, we have uploaded our datasets and Python code to an online GitHub repository [19].

## Materials and Methods

### Neural network training details

We use the Adam Optimizer [38], as implemented in TensorFlow (Keras) [18], for all training. We train using early stopping, saving only the best performing model, as measured by the validation loss on the development set. During training, we use learning rate annealing. Here, the annealing rate refers to the factor that we multiply the learning rate by at the beginning of each annealing step.

When training the forward neural network, we anneal across 4 steps with an annealing rate of 0.35, using an initial learning rate of 10^−3^ and an early stopping patience of 50. We use a minibatch size of 128 when training the forward network. When pretraining the inverse neural network, we anneal across 2 steps, each with no more than 50 epochs, using an initial learning rate of 2.5 × 10^−4^, an annealing rate of 0.6, and an early stopping patience of 5. After pretraining, we fine-tune the inverse neural network on the original training set used to train the forward network. In this stage, we anneal the inverse neural network across 3 steps with an annealing rate of 0.9. We use an initial learning rate of 2 × 10^−5^ and an early stopping patience of 40. When we train the inverse neural network without pretraining, as in Fig. 4A and B, we use an initial learning rate of 10^−3^, an annealing rate of 0.8, 10 annealing steps, and a patience of 40 for early stopping. We always use a minibatch size of 256 when training the inverse network.

The pretraining dataset uses 100,000 devices when training the inverse neural network to fit data from 2D transistors (e.g., Figs. 4 and 5). When training to fit data from HEMTs in Section S8, the size of the pretraining dataset is the smaller of (i) 100× the number of compact model simulations or (ii) 1,000,000. Additional computational details, including details for preprocessing and scaling, are described in Section S4.

### Experimental details for monolayer WS_2_ transistors

Monolayer WS_2_ was grown by hybrid metal–organic chemical vapor deposition and was wet-transferred onto 100-nm SiO_2_/p^++^ Si, which served as the back-gate. Polystyrene was spin-coated on top of the WS_2_ and then transferred in deionized water onto the target substrate (SiO_2_/Si). Polystyrene was then removed using toluene. Electron-beam lithography was employed for each lithography step. First, large probing pads (Ti/Pt 2/20 nm) were patterned and deposited by electron beam evaporation via lift-off. Channel definition was done using xenon difluoride etching, and the contacts were patterned for liftoff. Ni/Au contacts were evaporated by electron beam at ~10^−8^ torr. The channel and contact lengths of all devices were 500 nm and 1.5 μm, respectively. The devices were measured in a Janis ST-100 probe station at ~10^−4^ torr using a Keithley 4200 semiconductor parameter analyzer. These devices and measurements were described in a previous work [31].

Experimental devices were fabricated across 3 separate chips. We include all measured working devices in our test set, except for one device that could not achieve an on-state current >1 μA/μm at **V*_gs_ = 50 V andV*_ds_ = 1 V, leaving a total of 51 devices in our test set. Experimentally, we measure both forward and backward sweeps for each device using a *V*_gs_ range of ±60 V; in this work, we use only the forward sweeps and restrict *V*_gs_ from −6 to +50 V for the reverse-engineering process described in the main text.

## Data Availability

Sample training and test data used in this study are available in an online GitHub repository [19]. All other data that support the findings of this study are available from the corresponding author upon request. Code that supports the findings of this study is available in an online GitHub repository [19].

## Supplementary Materials

Supplementary 1 Sections S1 to S8 Figs. S1 to S6 Tables S1 and S2
