# Supplementary material for: Deep Learning to Automate Parameter Extraction and Model Fitting of Two-Dimensional Transistors
Source: Research (Wash D C). 2026 Aug 25;9:1103. doi: 10.34133/research.1103 (PMC13504040; doi:10.34133/research.1103)
Supplement: Supplementary file 1 [file research.1103.f1.pdf]

Supplementary Information

Deep Learning to Automate Parameter Extraction and Model Fitting of Two-Dimensional Transistors

Robert K. A. Bennett,[1] Jan-Lucas Uslu,[2,3] Harmon F. Gault,[1] Asir Intisar Khan,[1] Lauren Hoang,[1] Tara Peña,[1] Kathryn Neilson,[1] Young Suh Song,[1] Zhepeng Zhang,[3,4] Andrew J. Mannix,[3,4] Eric Pop[1,3,4,5,*]

[1]Department of Electrical Engineering, Stanford University, Stanford, CA 94305, USA
[2]Department of Physics, Stanford University, Stanford, CA 94305, USA
[3]Stanford Institute for Materials and Energy Sciences, SLAC National Accelerator Laboratory, Menlo Park, CA 94025, USA
[4]Department of Materials Science and Engineering, Stanford University, Stanford, CA 94305, USA
[5]Department of Applied Physics, Stanford University, Stanford, CA 94305, USA
*Contact: epop@stanford.edu

Supplementary Section 1: Details for Sentaurus simulations of two-dimensional transistors

We use Sentaurus Device,[1] an industry-standard technology computer-aided design (TCAD) simulator, for the physics-based simulations used in this work. We perform simulations in a two-dimensional domain using the cross-section shown in **Figure 2A** from the main text, where the width dimension of the device (into the page, perpendicular to the channel) is treated as periodic. Here, the default drift-diffusion model for transport is solved self-consistently with electrostatics (Poisson's equation).

We treat the two-dimensional (2D) semiconductor/metal contacts as Schottky contacts using Sentaurus Device's Schottky barrier model, and we enable tunneling through the barrier using Sentaurus Device's Schrodinger model on a nonlocal mesh. Although Fermi level pinning is known to affect the barrier height of 2D semiconductor/metal contacts,[2] Sentaurus Device does not presently have an established model for this pinning. To achieve a specific nominal barrier height, we therefore use the 2D semiconductor's electron affinity as a free parameter, with the resultant barrier height given by $\phi_{B0} = \chi_s - \phi_m$, where χ_s is the 2D semiconductor's electron affinity and ϕ_m = 5.01 eV is the work function of nickel. Gate-voltage-dependent contact resistance, an important experimental consideration,[3] is incorporated in our Sentaurus Device simulations automatically, as the electric field from the back-gate electrode electrostatically influences the 2D semiconductor underneath the metal.

We assign the monolayer 2D semiconductor an electronic band gap of 2.2 eV (larger than the optical band gap dictated by the exciton binding energy),[4,5] a channel thickness of 0.62 nm,[6] and in-plane and out-of-plane relative permittivities of 14 and 7,[7] respectively. We approximate the density of states using Sentaurus Device's effective mass model (as implemented by setting Formula=2).[1] We treat donor-like states (often attributed to chalcogen defects in a 2D semiconductor like WS_2) as states located at the 2D semiconductor/oxide interface, and we assume band-tail acceptor-like states are uniformly distributed across the 2D channel thickness.

Supplementary Section 2: Descriptions of model parameters for two-dimensional transistors

In **Table 1** of the main text, we list our fitting parameters alongside their lower and upper bounds. Here, we briefly explain the physical significance of each parameter and justify these bounds.

Mobility: A semiconductor's electron mobility is the proportionality constant between the electron speed and the applied electric field, assuming drift-diffusion transport (which is valid for the relatively long 500 nm channel lengths considered in this work). Typical academic 2D transistors often yield a mobility of the order ~10 $cm^2 V^{-1} s^{-1}$; here, we use a range of 1 to 35 $cm^2 V^{-1} s^{-1}$ to encapsulate this typical value. We note that electron mobilities above 35 $cm^2 V^{-1} s^{-1}$ are certainly possible in optimized devices with fewer defects; to fit such devices, the training set should be expanded to incorporate these higher mobilities.

The mobility considered in this work implicitly lumps various sources of mobility degradation (e.g., grain boundary scattering, remote phonon or charged impurity scattering) into one unified mobility. With appropriate mobility models, these factors could be included within the set of fitting parameters in **Table 1** to study these mechanisms further in emerging semiconductor devices.

Schottky barrier height: The Schottky barrier height is the energy barrier which a carrier must overcome to be injected from the metal contact to the semiconductor channel or vice versa. The Schottky barrier height is a function of its electrostatic environment and is hence not an intrinsic material or device parameter.[8] When we report Schottky barrier heights in this work, we specifically refer to the *nominal* Schottky barrier height $\phi_{B0} = \chi_s - \phi_m$, where χ_s is the semiconductor's electron affinity and ϕ_m is the metal contact's work function.

Contacts between 2D semiconductors and conventional metals are affected by Fermi level pinning, whereby metal-induced and/or defect-induced gap states cause the Fermi energy to become pinned at the charge neutrality level.[2] As a result, even though the work functions of metals and the electron affinities of two-dimensional semiconductors are well known, the real barrier height is not simply $\chi_s - \phi_m$. Processing conditions, the number of defects in the 2D semiconductor, strain profiles, and other non-idealities make accurately predicting this value difficult. Our previous experimental estimates for nickel + monolayer WS_2 systems put the barrier height between 170 and 400 meV;[9] here, we expand this range to 10 to 510 meV.

We note that because we do not explicitly model Fermi level pinning or other non-idealities, e.g., spatial variations of the Schottky barrier, the barrier height we extract represents an *effective* Schottky barrier height. To gain additional physical insights, one could develop a mapping of these extracted effective barrier heights to additional physical parameters (e.g., the Fermi pinning factor). Alternatively, one could directly incorporate additional non-idealities (such as Fermi level pinning) when building the TCAD training data set, possibly using parameterized functions involving new physical parameters, as a means of studying these non-idealities in more detail. Including these factors in simulations may increase the computational cost of building these training sets, so it could also be worthwhile to pretrain networks using data with effective barrier models and then fine-tune using more comprehensive simulations.

Density of states: a semiconductor's effective density of states N_C refers to the number of energy levels in which electrons can reside, with a larger density of states meaning that more electrons can become available

to conduct current (i.e., a higher density of states means higher conductance, assuming the same mobility). The density of states can be estimated from simulation techniques, such as density functional theory; however, local strain profiles, spacing of higher-energy valleys, and other extrinsic factors can affect a material's effective density of states. Typically, $N_C \approx 5\times10^{11}$ cm^{-2} for 2D semiconductors;[10] here, because of these uncertainties, we use a large expanded range of 2×10^{11} to 9×10^{12} cm^{-2} to comfortably encapsulate the expected N_C.

Donor defect profiles: experimental studies suggest that sulfur vacancies in two-dimensional semiconductors may act as electron donors.[11] Photoluminescence studies suggest that these vacancies follow Gaussian distributions with respect to energy, with shallow defects located a few tenths of an eV below the conduction band edge and deep states located nearer to the mid gap. Since we do not probe the deep subthreshold region in this work, we consider only the shallow defect states. A previous work[11] assigns these states a median energy $E_{D,mid} \approx 0.15$ eV below the conduction band edge and an energy width (i.e., standard deviation) $\sigma_D \approx 72$ meV (from Figure 3C of ref. [11]). See **Figure 2B** in the main text for a visual depiction of this profile and these parameters. Here, we use these as rough center points for our estimated ranges, sampling $E_{D,mid}$, and σ_D both between 20 – 200 meV. The peak concentration N_{D0} for these donor states can vary tremendously depending on film quality, making it difficult to estimate based on previous works. Here, we intentionally use a large range of $3\times10^{12} – 3\times10^{13}$ eV^{-1} cm^{-2} to encapsulate reasonable values for N_{D0}.

Band tail acceptor-like defect profiles: experimentally, 2D semiconductors do not have sharp conduction band edges; instead, the density of states decreases exponentially as E decreases below E_C. Although some works have investigated the influence of these band tail states on transport, the precise implications of band tails on electron transport in 2D semiconductors are unclear. In particular, the mobility of electrons contributed by band tail states is unclear, although it appears to be much less than that of conduction band electrons.

In this work, we adapt a convention previously applied for amorphous oxide semiconductors and model band tails as acceptor states and assign them 0 mobility.[12] This assumption is also made in the multiple-trap-and-release model, which is frequently used to describe transport in organic semiconductors.[13] Even though these states do not participate directly in conduction, their electrostatics are still considered explicitly: a higher density of tail states shifts the threshold voltage to the right and worsens subthreshold swing.

Previous work has estimated that the peak band tail density N_{A0} and characteristic energy decay length σ_A that characterize the exponential distribution [**Eq. (5)** in the main text] vary from $3\times10^{12} – 1.8\times10^{13}$ eV^{-1} cm^{-2} and from ~56 meV to 170 meV for similar 2D semiconductor systems.[14] Here, we expand these ranges to 6×10^{11} – 3.7×10^{13} eV^{-1} cm^{-2} and 50 – 300 meV, respectively, to comfortably encapsulate these initial estimates.

Supplementary Section 3: Feature engineering and ablation study

The inverse neural network shown in **Figure 3A** of the main text aims to reverse-engineer physics-based simulation model parameters from electrical measurements. For each device, we input drain current *vs*. gate-to-source voltage (I_d-V_{gs}) curves at 32 fixed V_{gs} values, with applied drain-to-source biases V_{ds} = 0.1 and 1 V. For each V_{ds}, we also perform simple feature engineering by inputting each current in logarithmic space, along with $\partial I_d/\partial V_{gs}$ and $\partial \log_{10} I_d/\partial V_{gs}$, for a total of eight unique features per device.

In principle, model parameters can be fit from experimental or simulated data using I_d-V_{gs} data at a single V_{ds}. Further, because derivatives and logarithms are implicitly contained within the data itself, seven of our eight features may seem unnecessary. Here, we justify from domain knowledge why one should expect that including these features should improve the performance of the neural network. After, we demonstrate empirically with an ablation study that failing to include these terms results in worse performance.

Including multiple drain biases: transistors with Schottky contacts, such as experimentally relevant two-dimensional transistors, can be conceptually thought of as an ideal ohmic transistor in series with two separate diodes that represent the source and drain.[15] These diodes are nonlinear circuit elements, meaning here that their resistances are functions of the voltage drops across them. Including multiple drain biases should help to ensure that the resistance of both the channel and the source/drain take on additional unique profiles, and thus aid in estimating the Schottky barrier height, one of our key fitting parameters.

Including logarithmic I_d-V_{gs} data: The off-state of the transistor provides essential information about key physical model parameters, including the acceptor and donor profiles that we parameterize using the five separate fitting parameters in **Table 1** in the main text. However, I_d in the off-state (low V_{gs}) can be orders of magnitude smaller than I_d in the on-state. For example, in **Figure 5A** in the main text, the ratio of on- to off-state current for many of our experimental WS_2 transistors is $>10^5$. If we did not take the logarithm of I_d, differences in off-state currents would be washed out, causing us to lose essential information in the data.

Including derivatives: mobility is commonly extracted from transistor measurements by finding the transconductance $g_m = \partial I_d/\partial V_{gs}$ and then estimating the mobility as $\mu = g_m/(C_g V_{gs} - V_T)$, where C_g is the gate capacitance. This mobility extraction is frequently made non-trivial by *contact gating*, i.e., the gate voltage modulates R_C, causing g_m to change from extrinsic factors, such as the Schottky barrier height.[3,16] Similarly, the subthreshold swing (inversely proportional to $\partial \log_{10} I_d/\partial V_{gs}$ in the off-state) can often be correlated to defect profiles [acceptor and donor, e.g., **Eqs. (4) and (5)** in the main text]. Thus, we explicitly input the derivatives to make these parameters easier for the neural network to extract.

Ablation study: we empirically verify the importance of including each feature by ablating them from the input matrix, (i.e., by setting all entries in the relevant feature columns to 0), and then re-training the neural networks as before. This process allows us to examine how withholding the feature impacts the performance of the final trained network. For each ablation, we use 1,000 devices in our training and development sets combined and perform 5 different runs, using the same 5 sets of training and development data for each ablation. Afterwards, we test the performance of the neural network by reverse-engineering the fitting parameters, estimating the corresponding I_d-V_{gs} curves for these extracted parameters, and then calculating the

R^2 fit between the original and estimated I_d-V_{gs} data. Here, all tests consider I_d-V_{gs} curves at V_{ds} = 0.1 and 1 V, where the R^2 fit is averaged in both linear and logarithmic space at both V_{ds} values (same as in the main text).

As shown in **Supplementary Figure S1A**, we find that the resultant I_d-V_{gs} fits become quite poor when we fail to include the logarithmic data or when we remove either drain bias. We find that the quality of the I_d-V_{gs} fits remain acceptable when we do not include the derivatives of the I_d-V_{gs} curves and when we include only the logarithmic data; however, as shown in **Supplementary Figure S1B**, the error in the fitting parameter increases if we exclude these features.

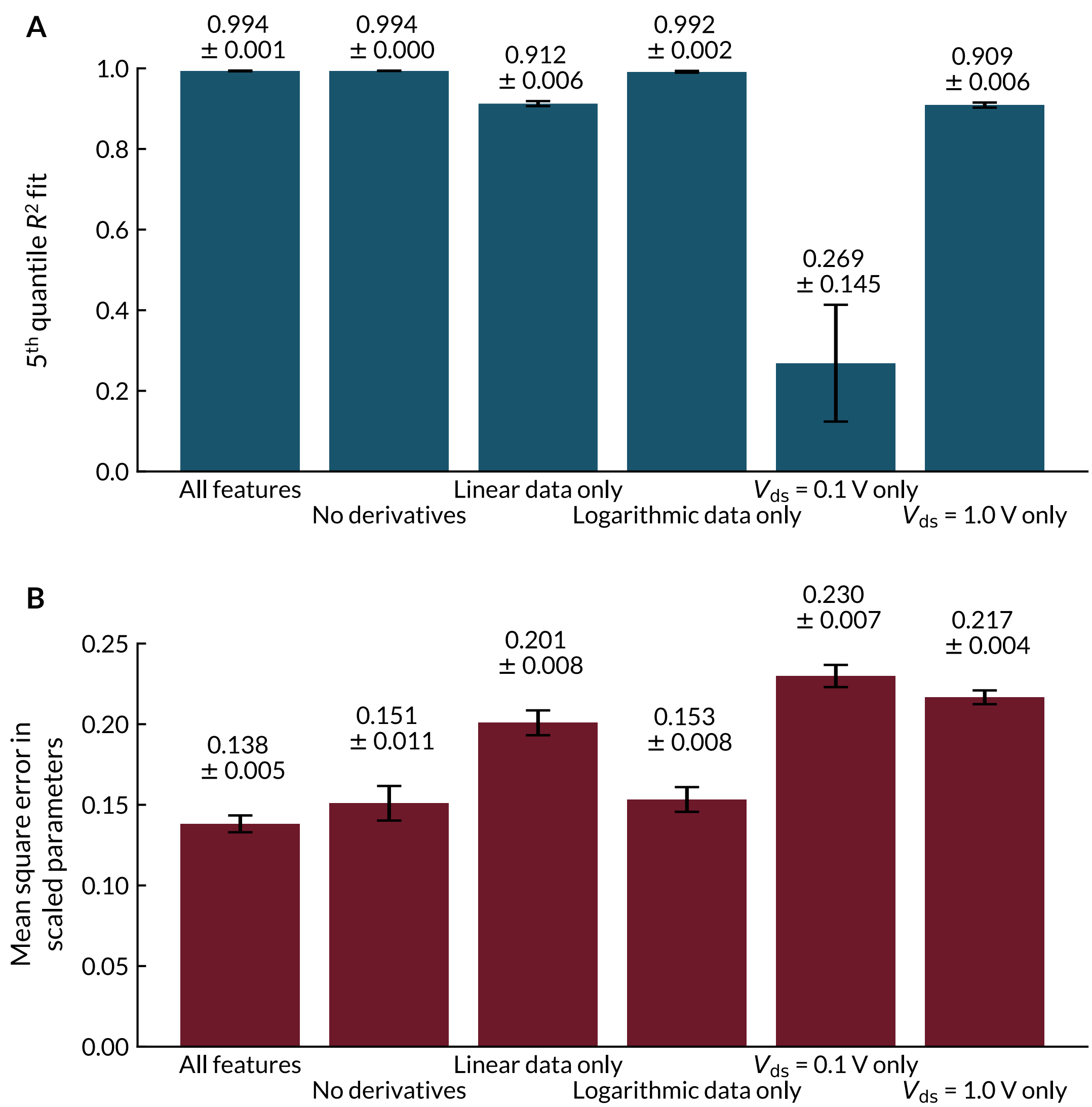

Supplementary Figure S1: Impact of ablating features on model performance. (**A**) The 5th quantile (i.e., worst 5%) R^2 fit (higher is better) and (**B**) mean square error in scaled parameters (lower is better) for different configurations of input features. We find that when removing our engineered features, the resultant fits become worse and the error in the fitting parameters increases. “Linear data only” and “logarithmic data only” denote that only the linear-scale I_d-V_{gs} curves and their derivatives, or logarithmic-scale I_d-V_{gs} curves and their derivatives, respectively, were included in the input matrix.

Supplementary Section 4: Details for pre-processing, bootstrapping, and testing

Interpolation

Our forward neural network (**Supplementary Figure S2A**) outputs I_d-V_{gs} curves along 32 fixed V_{gs} values, evenly spaced from -6 to 50 V. (Our Sentaurus Device simulations stopped just outside of the V_{gs} range of -6 to 50 V; hence, we actually use a range of -5.9 to 49.9 V throughout this work.) Our inverse neural network (**Figure 3A** in the main text) accepts I_d-V_{gs} curves and their derivatives along the same V_{gs} points. In our Sentaurus Simulations, we compute drain current *vs*. gate-to-source voltage (I_d-V_{gs}) profiles across a similar range of V_{gs} values; however, the exact V_{gs} grid changes between simulations to maximize computational efficiency and hence does not align perfectly with the 32 fixed V_{gs} values in the network's output and input layers. Therefore, we pre-process our simulated I_d-V_{gs} data by interpolating I_d along the fixed V_{gs} points required by our neural networks, using linear and logarithmic interpolation to interpolate I_d and $\log_{10} I_d$, respectively. This interpolation scheme and all pre-processing steps are implemented in process_devices.py in our GitHub Repository.[17]

Noise floor

Experimentally, the lowest drain current that can be reliably measured is limited by the *noise floor* of the measurement instrument. For our experimental monolayer WS_2 transistor measurements, our noise floor is ~10^{-5} µA/µm (**Figure 5A** in the main text). Our Sentaurus Device simulations allow us to simulate I_d much less than 10^{-5} µA/µm; however, we do not wish to train our neural networks to rely upon features that we do not measure experimentally. Thus, we manually impose a noise floor of 5×10^{-5} µA/µm on all Sentaurus-simulated data; that is, any $I_d < 5\times10^{-5}$ µA/µm is manually mapped to 5×10^{-5} µA/µm. To ensure that the noise floor in our simulated training/test sets and experimental test sets are identical, we perform the same mapping on our experimental I_d-V_{gs} curves prior to feeding them into our inverse neural network for inference.

Data formatting and min-max scaling

The inverse neural network accepts an input matrix for each device consisting of I_d-V_{gs} and $\log_{10}(I_d)$-V_{gs} curves and their derivatives (see input in **Figure 3A**), where columns correspond to individual features [e.g., I_d, $\log_{10}(I_d)$, $\partial I_d/\partial V_{gs}$] and rows correspond to the 32 fixed V_{gs} points. For training, these matrices for multiple devices are combined into a third order tensor $\boldsymbol{U}$. The first dimension of $\boldsymbol{U}$ corresponds to the device index, the second dimension corresponds to the 32 fixed V_{gs} points, and the third dimension corresponds to an individual feature. Here, we index starting from 0, and a colon (:) denotes selecting every entry along an index. For example, the slice $\boldsymbol{U}$[j, :, 0] is the I_d-V_{gs} curve at V_{ds} = 0.1 V for the jth device.

We prepare the scaled tensor $\widetilde{\boldsymbol{U}}$ by min-max scaling $\boldsymbol{U}$ feature-wise between -1 and 1. Each ith matrix slice along the last dimension of $\boldsymbol{U}$ is scaled independently using the minimum and maximum values across that individual slice. That is, for each ith slice along the third dimension,

$$\widetilde{\boldsymbol{U}}[:,:,i] = 2\left(\frac{\boldsymbol{U}[:,:,i] - \min(\boldsymbol{U}[:,:,i])}{\max(\boldsymbol{U}[:,:,i]) - \min(\boldsymbol{U}[:,:,i])}\right) - 1$$

Next, we consider the output of the forward neural network, $\boldsymbol{V}$, which is also a third-order tensor. $\boldsymbol{V}$ is a subset of $\boldsymbol{U}$ [$\boldsymbol{U}$ contains I_d-V_{gs} and $\log_{10}(I_d)$-V_{gs} curves and their derivatives at two drain biases; $\boldsymbol{V}$ contains the I_d-V_{gs} and $\log_{10}(I_d)$-V_{gs} curves, but not their derivatives]. Thus, $\boldsymbol{V}$ and its scaled equivalent $\widetilde{\boldsymbol{V}}$ are built simply by taking the required slices from $\boldsymbol{U}$ and $\widetilde{\boldsymbol{U}}$.

For the ith device, the input of the forward neural network and output of the inverse neural network is the vector $\boldsymbol{y}^{(\mathbf{i})}$. For training, these vectors are stacked into the matrix $\boldsymbol{Y}$, where the first dimension (rows) corresponds to the device index, and the second dimension (columns) corresponds to the fitting parameter. For example, $\boldsymbol{Y}[i,j]$ corresponds to the jth fitting parameter of the ith device.

We min-max scale $\boldsymbol{Y}$ between -1 and 1 for each fitting parameter:

$$\widetilde{\boldsymbol{Y}}[:,j] = 2\left(\frac{\boldsymbol{Y}[:,j] - \min(\boldsymbol{Y}[:,j])}{\max(\boldsymbol{Y}[:,j]) - \min(\boldsymbol{Y}[:,j])}\right) - 1$$

All min-max scaling is implemented in process_devices.py in our GitHub Repository.

Calculating the current mismatch in the inverse neural network loss function

In **Eq. (6)** of the main text, we introduce the term $E_{I_\text{d}}^{(i)}$ to allow the loss function to penalize errors in the predicted I_d-V_{gs} curves for the ith device. Here, $E_{I_\text{d}}^{(i)}$ is the sum of three terms that individually quantify errors in the I_d-V_{gs} curves, their slopes, and their curvatures:

$$E_{I_\text{d}}^{(i)} = {L_{I_\text{d}}^{(i)}}^2 + {L_{\Delta I_\text{d}}^{(i)}}^2 + {L_{\Delta^2 I_\text{d}}^{(i)}}^2 \tag{S1}$$

where:

$${L_{I_\text{d}}^{(i)}}^2 = \frac{1}{2N_{V_\text{ds}}N_{V_\text{gs}}}\sum_j^{N_{V_\text{ds}}}\sum_k^{N_{V_\text{gs}}}\left(\left(I_\text{true}^{(i,j,k)} - I_\text{pred}^{(i,j,k)}\right)^2 + \left(\log_{10} I_\text{true}^{(i,j,k)} - \log_{10} I_\text{pred}^{(i,j,k)}\right)^2\right) \tag{S2}$$

$${L_{\Delta I_\text{d}}^{(i)}}^2 = \frac{1}{2N_{V_\text{ds}}\left(N_{V_\text{gs}}-1\right)}\sum_j^{N_{V_\text{ds}}}\sum_k^{N_{V_\text{gs}}-1}\left(\left(\Delta I_\text{true}^{(i,j,k)} - \Delta I_\text{pred}^{(i,j,k)}\right)^2 + \left(\Delta \log_{10} I_\text{true}^{(i,j,k)} - \Delta \log_{10} I_\text{pred}^{(i,j,k)}\right)^2\right) \tag{S3}$$

$${L_{\Delta^2 I_\text{d}}^{(i)}}^2 = \frac{1}{2N_{V_\text{ds}}\left(N_{V_\text{gs}}-2\right)}\sum_j^{N_{V_\text{ds}}}\sum_k^{N_{V_\text{gs}}-2}\left(\left(\Delta^2 I_\text{true}^{(i,j,k)} - \Delta^2 I_\text{pred}^{(i,j,k)}\right)^2 + \left(\Delta^2 \log_{10} I_\text{true}^{(i,j,k)} - \Delta^2 \log_{10} I_\text{pred}^{(i,j,k)}\right)^2\right) \tag{S4}$$

where N_{V_ds} and N_{V_gs} are the number of V_{ds} and V_{gs} values, respectively, and j and k denote the jth V_{ds} and k^{th} V_{gs} value, respectively. Δ and Δ^2 denote first and second differences with respect to V_{gs}, e.g.,

$$\Delta I_\text{true}^{(i,j,k)} = I_\text{true}^{(i,j,k)} - I_\text{true}^{(i,j,k-1)} \tag{S5}$$

$$\Delta^2 I_\text{true}^{(i,j,k)} = \Delta I_\text{true}^{(i,j,k)} - \Delta I_\text{true}^{(i,j,k-1)} \tag{S6}$$

Bootstrapping studies

We use a bootstrapping approach in **Figures 4A,B** in the main text to understand how varying the training set size influences the neural network's performance when reverse-engineering two-dimensional transistors. For each bootstrapped simulation, we build smaller training subsets by randomly selecting the specified number of devices from the original I_d-V_{gs} simulations. Afterwards, we initialize the forward and inverse models with random weights using the Glorot uniform initializer as implemented in TensorFlow, and carry out the training scheme using these smaller training sets.

R^2 calculations

Throughout the main text and supplementary information, we report individual R^2 values to measure the goodness of fit between data sets containing multiple baseline truths and predictions. A conventional R^2 that summarizes multiple data sets is not typically well defined. Here, for a set of N curves, we calculate the individual R^2 for every curve and then take the average of these values. For our I_d-V_{gs} curves, we include both the R^2 in linear space (i.e., as normally calculated) and R^2 in logarithmic space (i.e., transforming both the baseline truth and predicted I_d by taking their base 10 logarithm and then calculating the R^2 with these transformed values) in the above sum. These R^2 values are calculated on baseline and predicted I_d-V_{gs} curves after interpolating them onto 32 evenly spaced V_{gs} points and after imposing noise floor mapping, as described in the "interpolation" and "noise floor" procedures earlier in this section.

Supplementary Section 5: Details for the forward neural network

Here, we discuss the *forward neural network* (architecture in **Supplementary Figure S2A**) used to predict I_d-V_{gs} curves from the model parameters in **Table 1**. In essence, this neural network approximates the original physics-based TCAD solver or, equivalently, it approximates $\boldsymbol{M}_{\mathbf{forward}}$ in **Eq. (1)**. We emphasize that the forward neural network should not be confused with the *inverse neural network* that we use to reverse-engineer model parameters; the forward neural network is a surrogate model that (i) provides an augmented data set for pretraining our inverse neural network and (ii) is called when evaluating the loss function of our inverse neural network using a tandem approach (as in **Figure 3B** in the main text).

After scaling all data, we train the forward neural network to output I_d-V_{gs} curves with V_{gs} values evenly spaced from -6 to +50 V (see **Supplementary Section 4** for scaling and preprocessing procedures). The network outputs I_d-V_{gs} curves at V_{ds} = 0.1 and 1.0 V, in both linear and logarithmic space, yielding four separate curves. Although predicting both I_d and $\log_{10}(I_d)$ explicitly may seem redundant, we do so because the hyperbolic tangent activation functions in the network's output layer have range (-1, 1) and hence cannot easily predict outputs that span multiple orders of magnitude. Thus, we explicitly output $\log_{10}(I_d)$ to ensure we can accurately predict the off-state current, which tends to be many orders of magnitude smaller than the on-state current.

Forward neural network loss function

We use the loss function shown in **Eq. (S7)** when training the forward neural network. Here, we aim to ensure that the forward neural network predicts both the linear and $\log_{10}I_d$ values, along with the slopes and curvatures of the linear and $\log_{10}I_d$-V_{gs} curves, across all V_{ds} values.

$$\mathcal{L}_{\text{forward}} = \frac{1}{N_{\text{devices}}} \sum_{i}^{N_{\text{devices}}} \left({L_{I_\text{d}}^{(i)}}^2 + {L_{\Delta I_\text{d}}^{(i)}}^2 + {L_{\Delta^2 I_\text{d}}^{(i)}}^2 \right) \tag{S7}$$

Here, $N_{devices}$ is the number of devices in the training set and i denotes the ith device. Other terms in **Eq. (S7)** quantify the error in the I_d-V_{gs} measurements, along with errors in their slopes and curvatures, and are defined in **Eqs. (S2) to (S4)**. Note that, unlike in **Eq. (6)** in the main text, we do not take the square root of the bracketed term in the forward neural network's loss function.

Forward neural network performance

Because physics-based (TCAD) I_d-V_{gs} training data are computationally expensive, we wish to obtain high quality fits using as little training data as possible. Thus, we assess the forward network's performance *vs*. the number of devices included in the training set using the bootstrap procedure described in **Supplementary Section 4.** Here, we find that for all training set sizes considered, our forward neural network can achieve a median $R^2 \geq 0.98$ on the test set (**Supplementary Figure S2B**), indicating a good match between the original simulated current and the approximation from the forward neural network. We are also interested in the network's performance across the vast majority of the test set; thus, we plot the 5th quantile R^2 (i.e., the R^2 corresponding to the worst 5% of fits) on the same axes, and we plot sample cumulative histograms of R^2 for different training set sizes in **Supplementary Figure S2C**. We find that the worst 5% of the fits still match well when the training set contains at least ~500 devices, for which we achieve a 5th quantile $R^2 \approx 0.94$. We show sample fits corresponding to the median R^2 after training on 250, 1,000, and 4,000 devices in

Supplementary Figures S2D-F to confirm agreement between the original physics-based simulation and the network's predicted I_d.

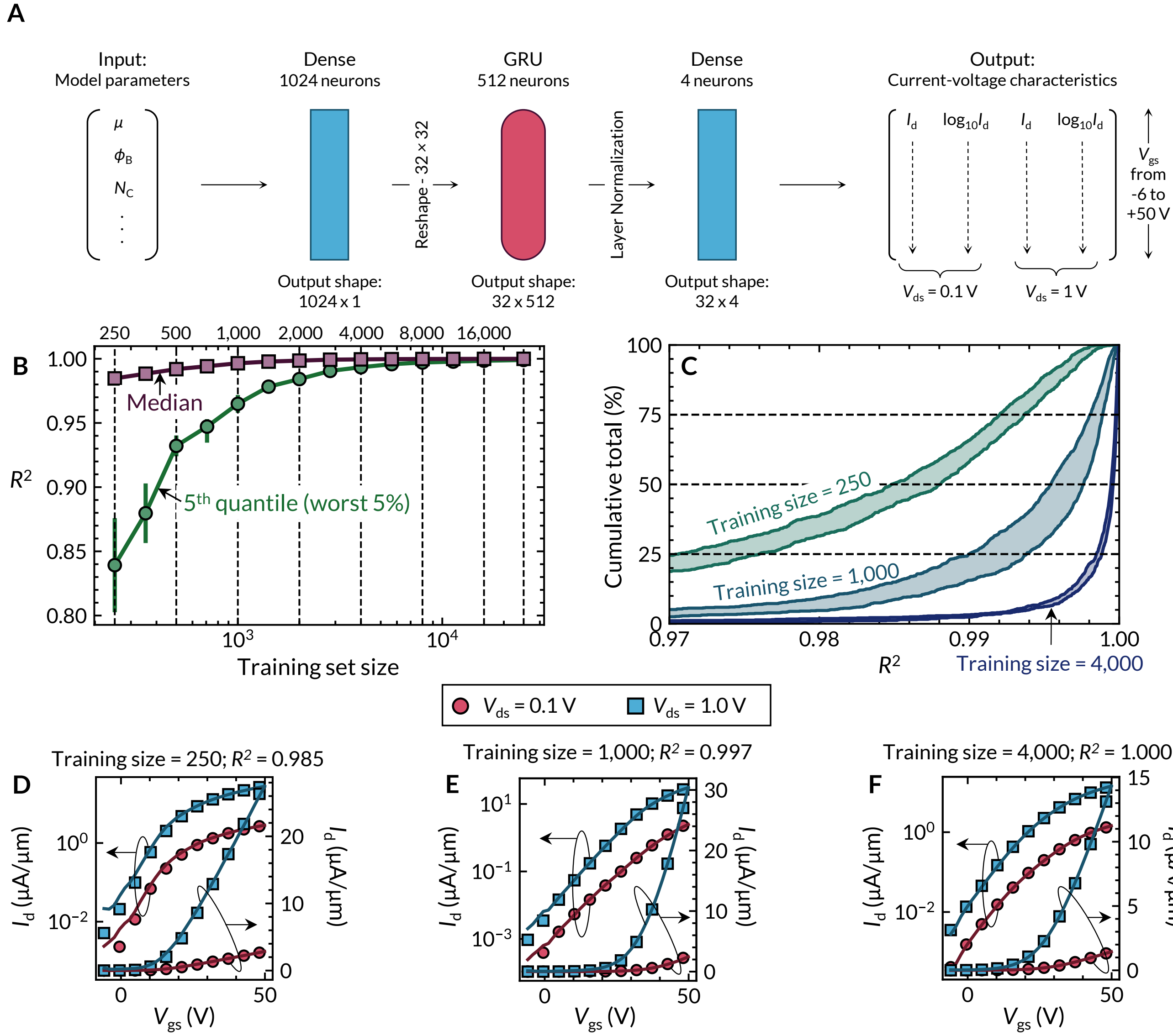

Supplementary Figure S2: Performance of the forward neural network used as a surrogate model. (**A**) The architecture of the neural network used to approximate the physics-based current model ("the forward neural network"). Dense layers use the hyperbolic tangent (tanh) activation function. The gated recurrent unit (GRU) layer uses tanh for its activation function and sigmoid for its recurrent activation function. (**B**) Median and 5th quantile (i.e., worst 5%) R^2 fits between the I_d calculated from the physics-based simulation and estimated by the forward neural network *vs.* the number of devices in the training set. Model fits were evaluated using a test set of 1,000 devices that were unseen by the model during training and network optimization. Error bars show standard deviation across five bootstrap tests (see **Supplementary Section 4** for details); error bars are often extremely small and hence are not always visible. (**C**) Cumulative R^2 fits for various training set sizes, where shaded regions show the range for the cumulative R^2 across the three bootstrapped tests. Current *vs*. gate-to-source voltage plots corresponding to the median R^2 fits after training on (**D**) 250 devices, (**E**) 1,000 devices, and (**F**) 4,000 devices (each plotted for the worst-performing bootstrapped network). Note that each device shown is different, because they are generated from a distribution of input parameters as listed in **Table 1**. Symbols: original physics-based simulations; lines: neural network predictions. The data are shown on both logarithmic (left) and linear axes (right), at V_{ds} = 0.1 V (red symbols) and 1.0 V (blue symbols).

To assess the performance of the inverse neural network on the Sentaurus-simulated test set in **Figure 4** of the main text and during our ablation study in **Supplementary Section 3**, we compare the original I_d-V_{gs} curves to those obtained using the reverse-engineered parameters. To estimate the reverse-engineered I_d-V_{gs} curves, we take these extracted parameters and input them into a well-trained forward neural network. This forward network was trained on all 25,000 devices in our original physics-based training set and followed the same training procedure as described in **Supplementary Section 4**, with additional epochs and annealing steps to further minimize errors.

We test this well-trained forward network on the same test set of 1,000 devices and find that it reproduces the original Sentaurus-simulated I_d-V_{gs} characteristics exceptionally well: as shown in **Supplementary Figure S3**, this forward neural network achieves a 0.5th quantile, 1st quantile, and 2.5th quantile (i.e., worst 0.5%, worst 1%, and worst 2.5%) R^2 fit of 0.9915, 0.9970, and 0.9973, respectively, validating the use of this method for estimating R^2.

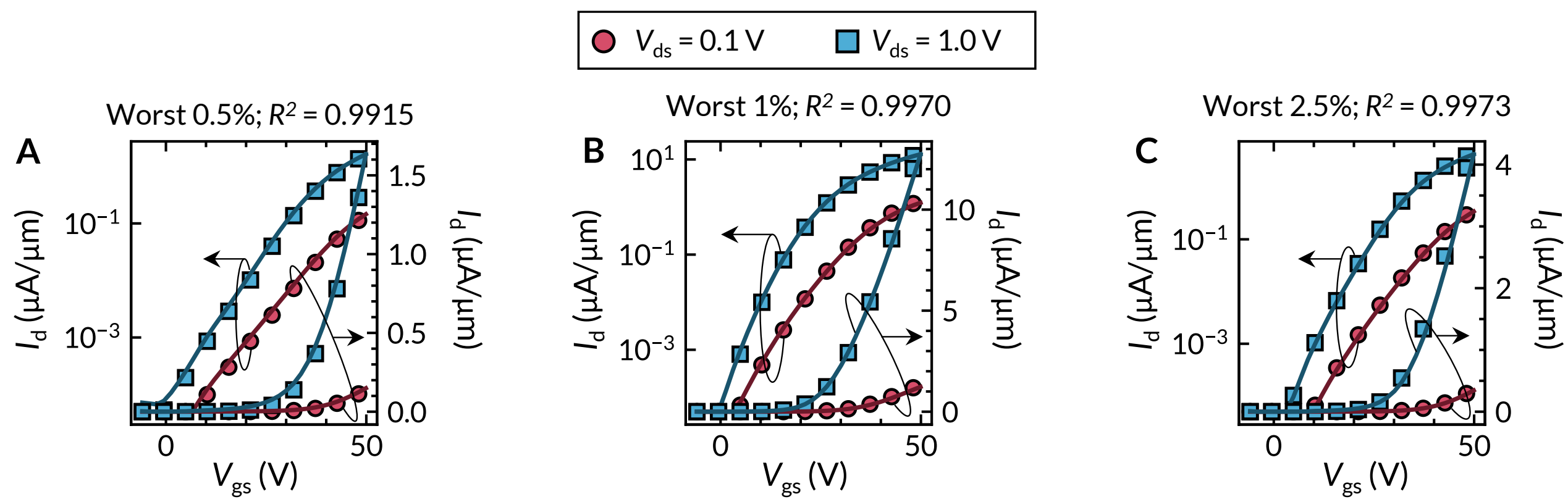

Supplementary Figure S3: Performance of the well-trained forward neural network used to estimate R^2 in Figure 4 of the main text. Current *vs*. gate-to-source voltage plots of the worst (**A**) 0.5%, (**B**) 1%, and (**C**) 2.5% R^2 sets obtained when testing the well-trained forward neural network on the Sentaurus-simulated test set. Even for these pessimistic scenarios, we find high $R^2 > 0.99$ across nearly all of the test set. Symbols: original physics-based simulations; lines: neural network predictions. The data are shown on both logarithmic (left) and linear axes (right), at V_{ds} = 0.1 V (red symbols) and 1.0 V (blue symbols).

Supplementary Section 6: Complete parameter extractions

In **Supplementary Figure S4**, we show the actual *vs*. predicted values and corresponding error histograms for the eight model parameters from **Table 1** in the main text. We extract these parameters for the test set of 1,000 Sentaurus-simulated devices using the same inverse neural network as used in **Figures 4C-H** in the main text (i.e., this neural network was trained using 500 devices included in the main physics-based training set). To avoid cluttering plots of actual *vs*. predicted data, we plot only 250 out of 1,000 points from the test set when plotting actual *vs*. predicted values.

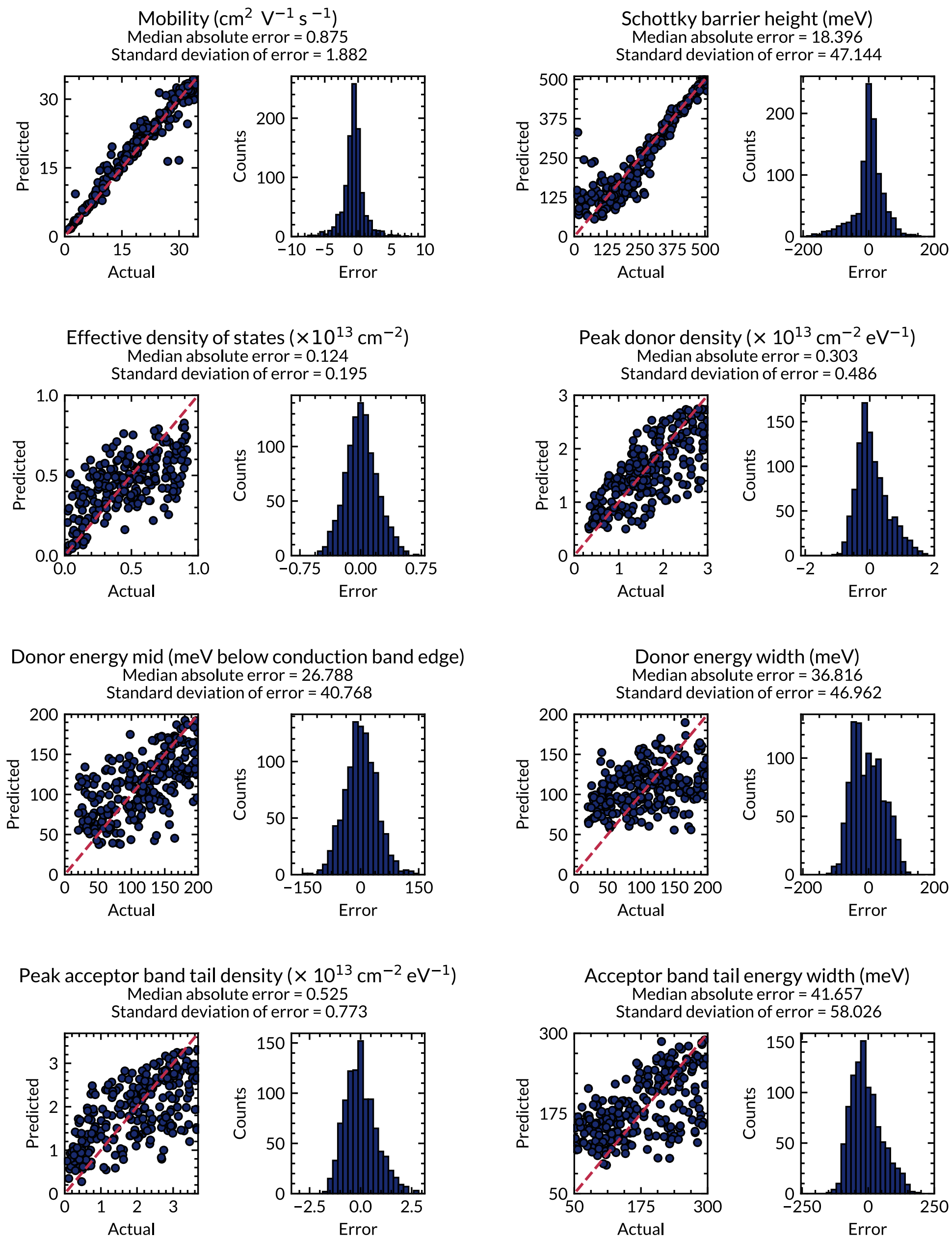

Supplementary Figure S4: Errors for individual parameters extracted from the simulated test set discussed in the main text. Each subpanel shows actual *vs*. predicted values and corresponding error histograms for the eight model parameters from **Table 1**. The median absolute errors and standard deviation of errors were calculated from the full 1,000 devices, and the histograms show data from all devices as well. All units for each plot, except for histogram counts, are as indicated in the corresponding subpanel titles. We truncate the *x*-axes of the histograms at ± ~4×the standard deviation of error for clarity.

When training new deep learning models for parameter extraction, we strongly recommend preparing similar statistical plots as in **Supplementary Figure S4** using synthetic test data to assess which parameters are especially error-prone. Once these plots are prepared, the accuracy of extracted parameters for an individual device can be estimated by examining the range of errors given the extracted values. For instance, if the deep learning approach estimates a Schottky barrier height $\phi_B \approx 500$ meV, the ϕ_B statistics from **Supplementary Figure S4** suggest that this estimate is likely quite accurate. Alternatively, if ϕ_B is estimated to be ~125 meV, then this figure suggests that the extracted value is more error-prone, likely being somewhere between ~0 and ~200 meV. This procedure could be repeated for every device parameter to achieve detailed distributions (e.g., histograms) estimating their uncertainty.

Another way to estimate the errors in extracted parameters for a particular device is to train multiple inverse neural networks from randomly initialized starting points. Then, one could use each of the bootstrapped networks to extract the parameter set for the same device multiple times. The value of each extracted parameter will vary across some range, with a narrow range indicating higher reliability. We note that the entire training procedure we implement here, including training the forward neural network, pretraining the inverse neural network, and fine-tuning the inverse neural network, takes less than 15 minutes on a single NVIDIA RTX 4080 graphics processing unit, making this analysis computationally tractable.

To examine how interplay between parameters could affect extraction errors, we examine the distribution of errors in the peak band tail density N_{A0} and acceptor band tail energy width σ_A as a test case. Together, these parameters define the acceptor density N_A *vs*. energy E profile, $N_A(E)$. From **Equation 5** in the main text, $N_A(E)$ increases with N_{A0} and decreases with σ_A; therefore, if one were to overestimate N_{A0}, then the overall $N_A(E)$ profile (the actual physical quantity of interest) could be partially recovered by decreasing σ_A to compensate, or vice-versa.

To confirm that our inverse neural networks do compensate for parameter errors in this way, we plot in **Supplementary Figure S5** the difference between the actual and predicted σ_A as a function of the error in N_{A0}, i.e., we plot $\sigma_A^{(\text{actual})} - \sigma_A^{(\text{pred})}$ *vs*. $N_{A0}^{(\text{actual})} - N_{A0}^{(\text{pred})}$ where the superscripts "actual" and "pred" denote actual and predicted quantities, respectively. Here, we find that there is indeed a clear trend between these errors; furthermore N_{A0} overestimation is accompanied by σ_A underestimation, or vice-versa, 77% of the time.

We note that there is likely similar interplay between all parameters; studying this interplay in detail would necessitate a thorough multivariate analysis that is beyond the scope of this work. Nevertheless, the correlation between errors shown in **Supplementary Figure S5** supports our hypothesis that some of the fitting uncertainty can be explained by interplay between parameters in our electrical simulations.

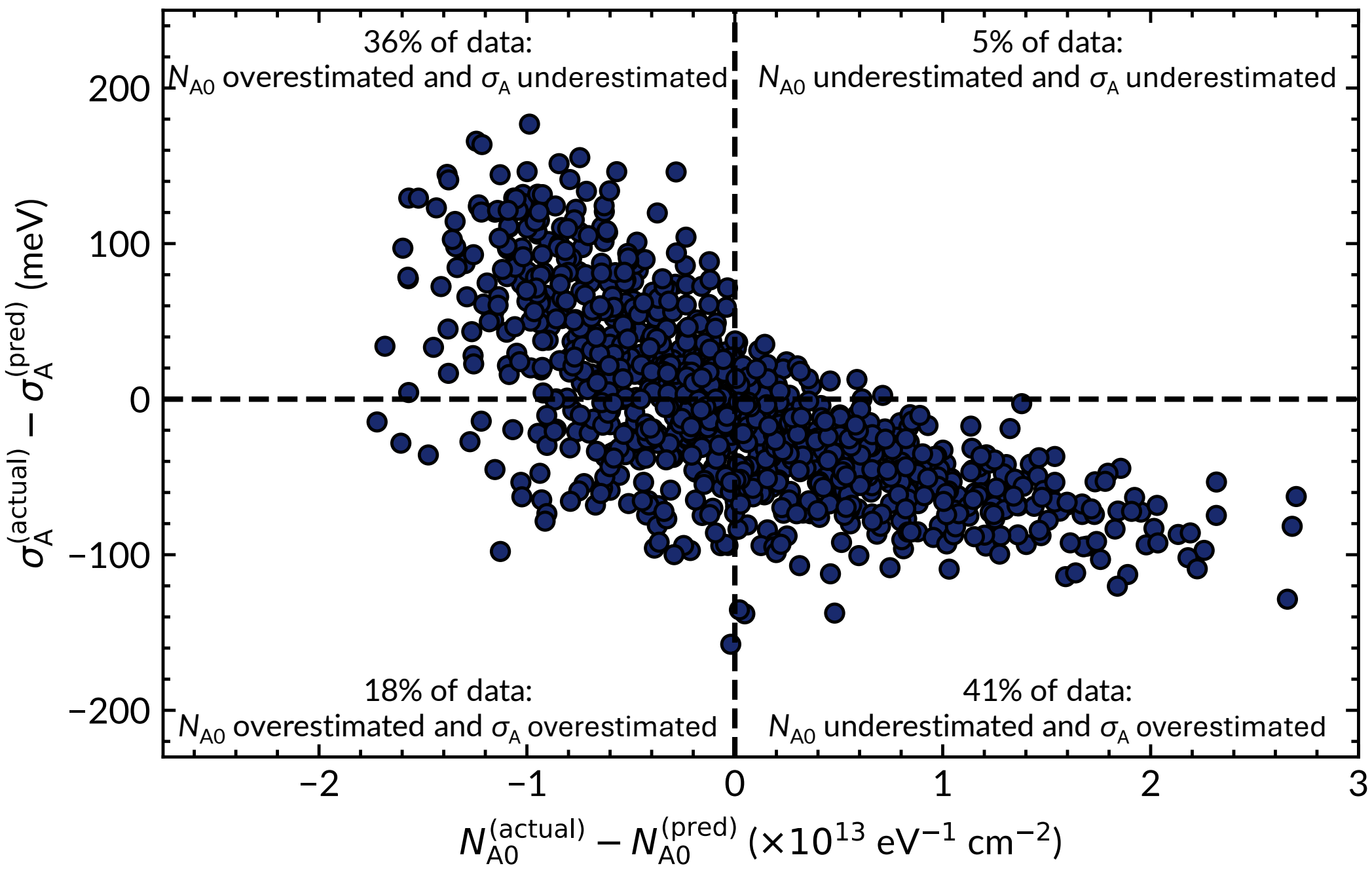

Supplementary Figure S5: Correlation between errors in parameters that define the total band-tail-like acceptor density. Here, we plot the error in the acceptor band tail energy width σ_A as a function of the error in the peak acceptor band tail density N_{A0}, i.e., $\sigma_A^{(actual)} - \sigma_A^{(pred)}$ vs. $N_{A0}^{(actual)} - N_{A0}^{(pred)}$, where the superscripts "actual" and "pred" denote actual and predicted quantities, respectively.

Supplementary Section 7: Fits for all experimental devices

Below, we show fits across the experimental test set of 51 monolayer WS_2 transistors, ordered by ascending R^2. We title each plot with the number of the device (ordered by the quality of the fit; 1/51 indicates the worst R^2 fit and 51/51 indicates the best R^2 fit). Filled markers and solid lines show the data on the linear scale (right) y-axis, and hollow markers and dashed lines show the data on the logarithmic scale (left) y-axis.

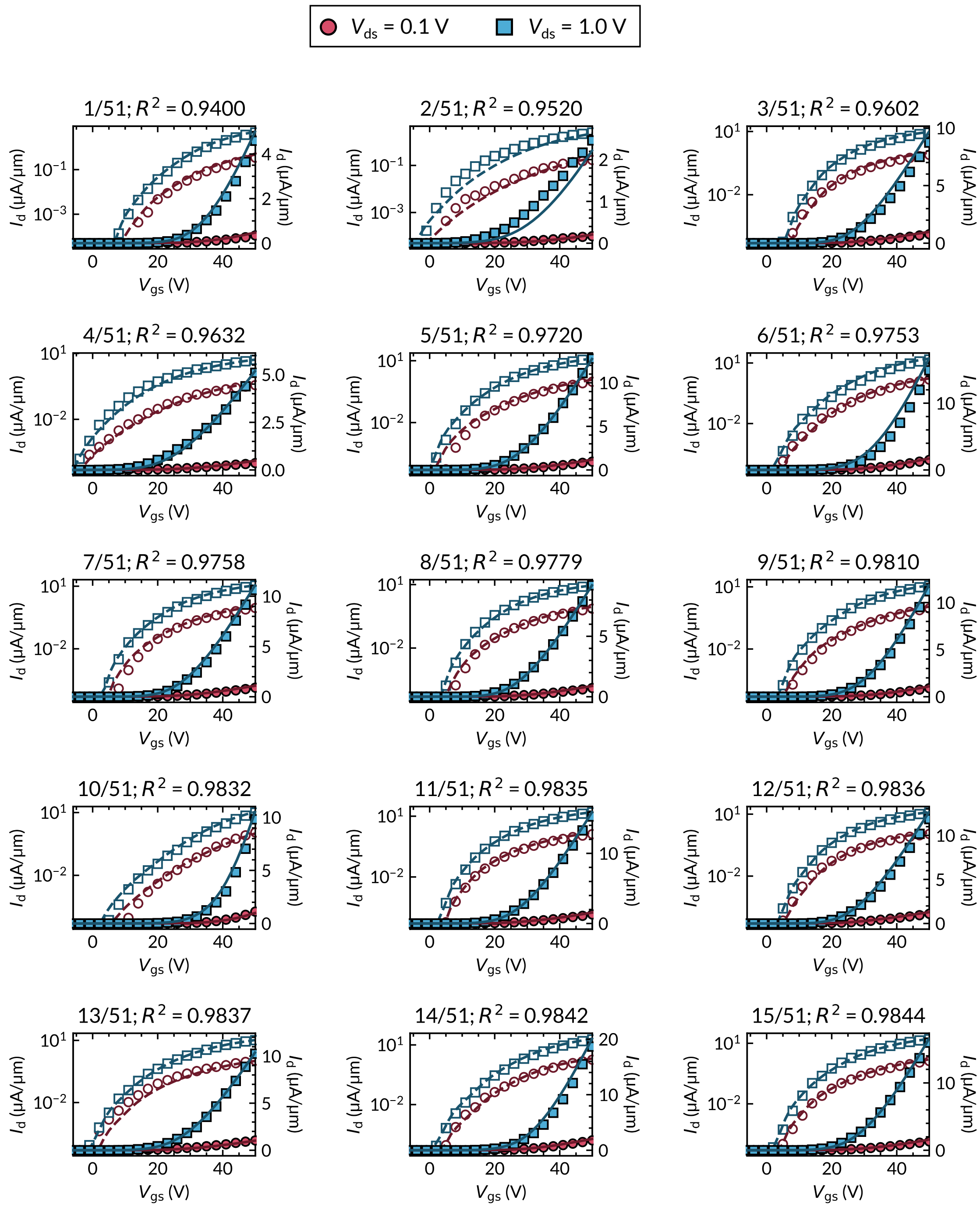

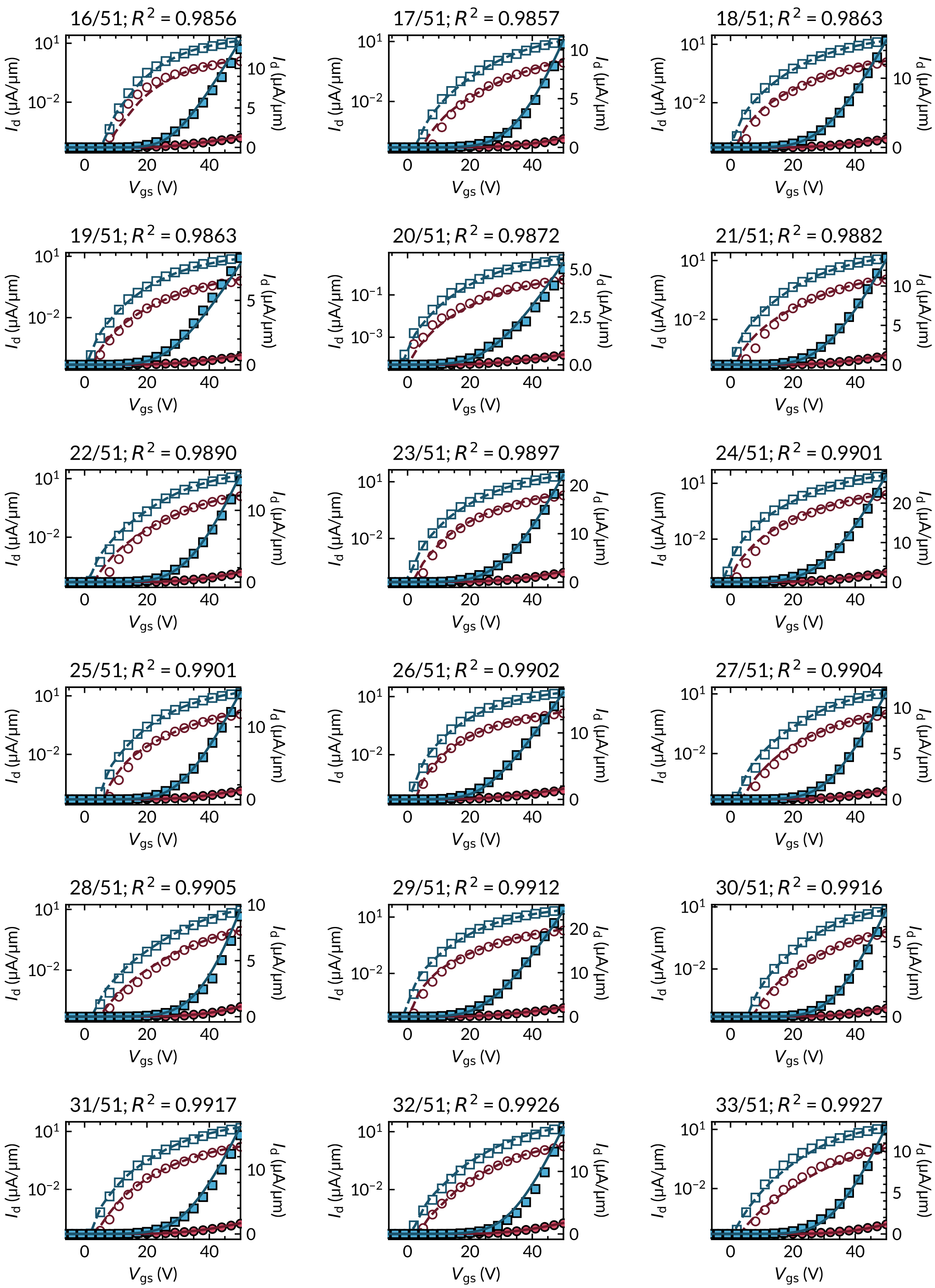
16/51; R² = 0.9856
17/51; R² = 0.9857
18/51; R² = 0.9863
19/51; R² = 0.9863
20/51; R² = 0.9872
21/51; R² = 0.9882
22/51; R² = 0.9890
23/51; R² = 0.9897
24/51; R² = 0.9901
25/51; R² = 0.9901
26/51; R² = 0.9902
27/51; R² = 0.9904
28/51; R² = 0.9905
29/51; R² = 0.9912
30/51; R² = 0.9916
31/51; R² = 0.9917
32/51; R² = 0.9926
33/51; R² = 0.9927
Id (μA/μm)
Vgs (V)

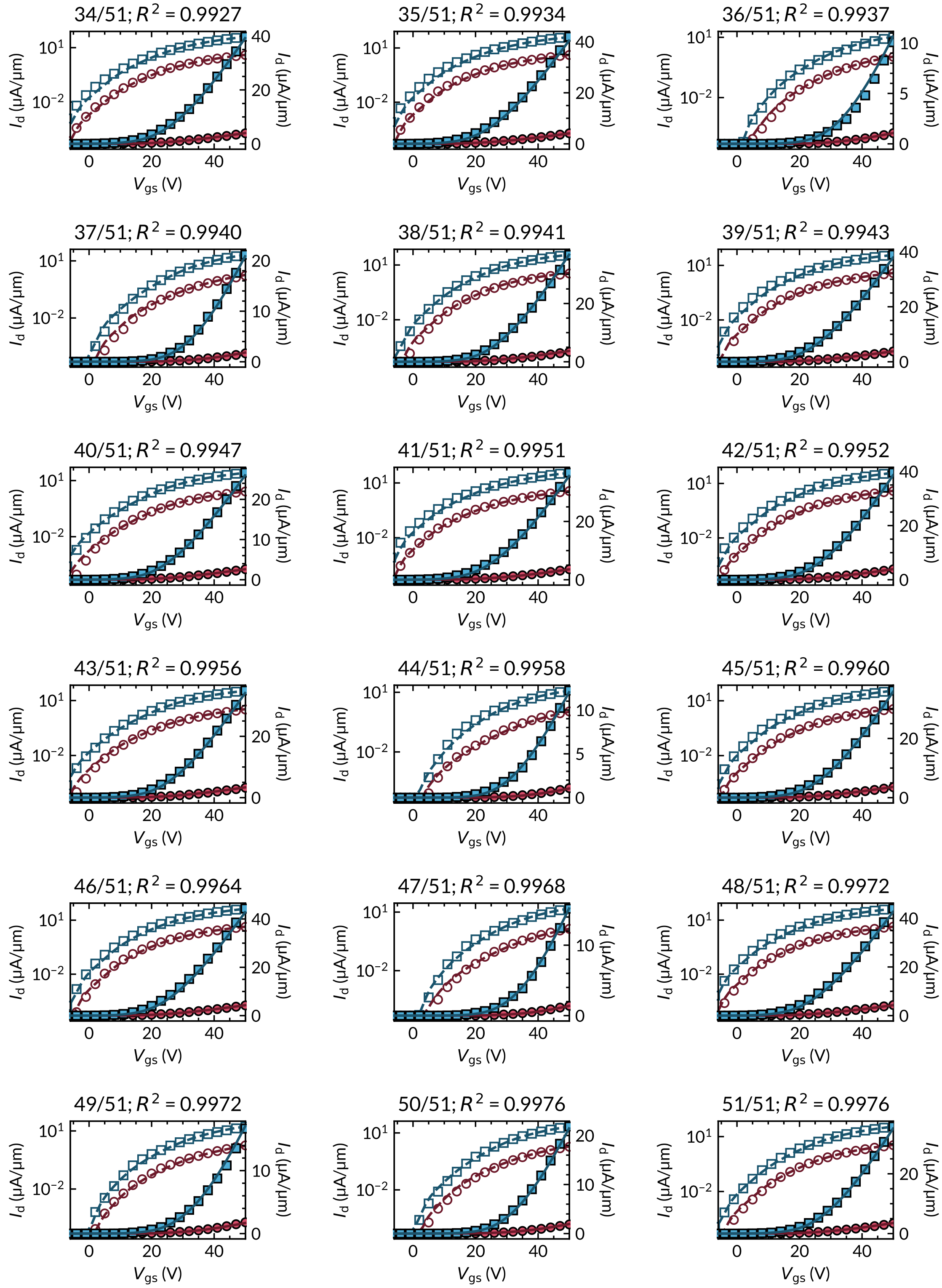
34/51; R^2 = 0.9927
35/51; R^2 = 0.9934
36/51; R^2 = 0.9937
37/51; R^2 = 0.9940
38/51; R^2 = 0.9941
39/51; R^2 = 0.9943
40/51; R^2 = 0.9947
41/51; R^2 = 0.9951
42/51; R^2 = 0.9952
43/51; R^2 = 0.9956
44/51; R^2 = 0.9958
45/51; R^2 = 0.9960
46/51; R^2 = 0.9964
47/51; R^2 = 0.9968
48/51; R^2 = 0.9972
49/51; R^2 = 0.9972
50/51; R^2 = 0.9976
51/51; R^2 = 0.9976
Id (μA/μm)
Vgs (V)

Supplementary Section 8: Details for high electron mobility transistor simulations

We use the fitting parameters and ranges shown in **Supplementary Table S1** for high-electron-mobility transistors (HEMTs) with 150 nm channel lengths. We build our training/test sets from the ASM-HEMT compact model,[18] and we pre-process in the same way as for two-dimensional transistors. Our training and test data sets consist of I_d-V_{gs} curves from V_{gs} = -3.5 to 0.5 V (evenly spaced across 32 values) at fixed drain biases of V_{ds} = -1, 0.1, 1, 5, 10, 15, and 20 V. All neural network architectures are nearly identical to those used for two-dimensional transistors, except that the forward neural network uses 512 and 256 neurons in its first dense layer and its gated recurrent unit (GRU) layer, respectively, and the number of neurons in the input and output layers are updated to accommodate the number of fitting parameters and V_{ds} values. Here, we wish to understand the impact of increasing the number of fitting parameters on the accuracy of the inverse design procedure. Thus, we bootstrap not only the training data, but also the fitting parameters themselves.

When varying the number of fitting parameters used, we select the parameters that we vary at random from **Supplementary Table S1**; all other parameters are assigned default values at random from their respective ranges. This parameter set is very similar to that used in a previous work,[19] with the ranges of some parameters having been expanded. We note that different fitting parameters could be easier or more difficult to fit compared to others. Therefore, for each of our five bootstrapped runs, we always progressively add additional fitting parameters. For example, in a bootstrap run, we begin by fitting 5 randomly selected parameters. When we fit 10 parameters, we select 5 additional parameters to vary alongside the first 5. When we fit 15 parameters, we select 5 additional parameters to vary alongside the first 10, and so on. For the next bootstrap run, we pick 5 new random fitting parameters and begin this process anew.

Supplementary Table S1: Fitting parameters and ranges across which they vary for the ASM-HEMT compact model simulations. The parameters and ranges across which they vary are very similar to those in a previous work[19], with some ranges expanded (parameters not listed here take on the default values from this same reference). All parameter names and units are identical to those in the ASM-HEMT model. All fitting parameters were sampled uniformly across their respective ranges. A superscripted asterisk (*) after the parameter name denotes that this sampling was performed in logarithmic (base 10) space, e.g., for a parameter with lower and upper bounds A and B, we drew a random number c sampled uniformly between $\log_{10}A$ and $\log_{10}B$ and assigned the parameter a value $y = 10^c$.

Parameter name	Range	Parameter name	Range
cdscd	0.01 – 0.15	rnjgd	15 – 30
eta0	0.01 – 0.1	rnjgs	5 – 15
delta	2 – 5	rsc	10^{-5} – 1.5×10^{-3}
gdsmin*	10^{-12} – 10^{-6}	rth0	15 – 45
igddio	7.5 – 15	thesat	1 – 4
igsdio	2.5 – 10	u0	0.15 – 0.3
imin*	10^{-15} – 10^{-12}	u0accd	0.05 – 0.25
lambda	10^{-4} – 5×10^{-3}	u0accs	0.05 – 0.25
mexpaccs	1 – 5	ua	10^{-8} – 5×10^{-7}
mexpaccd	1 – 5	ub*	10^{-21} – 10^{-15}
nfactor	0.2 – 0.5	ute	-1 – -0.1
njgd	2.5 – 20	uted	-17.5 – -5
njgs	2.5 – 20	utes	-17.5 – -5
ns0accd*	5×10^{15} – 5×10^{20}	vdscale	2 – 6
ns0accs*	5×10^{15} – 5×10^{20}	voff	-2.1 – -1.9
rdc	10^{-4} – 1.5×10^{-3}	vsat	1.5×10^{6} – 2.5×10^{6}
rigddio	10^{-8} – 10^{-7}	vsataccs	10^{4} – 1.5×10^{5}
rigsdio	10^{-8} – 10^{-7}		

We plot the 5th quantile (worst 5%), 10th quantile (worst 10%), and median R^2 fit as functions of the number of fitting parameters in **Supplementary Figures S6A-C** for various training size sets. Each test set contains sets of I_d-V_{gs} curves from 1,000 HEMTs that were unseen by the neural network during training and optimization. Here, we find that increasing the number of fitting parameters makes the extraction process more error-prone if the size of the training set remains constant. This is an unsurprising consequence of the "curse of dimensionality,"[20] since the available parameter space grows exponentially as the number of fitting parameters increases, making it increasingly difficult for the neural network to fit multiple parameters simultaneously. However, as we increase the size of our training sets, we find that we can somewhat compensate for this added complexity – for example, from **Supplementary Figure S6B**, we observe that when fitting 15 parameters simultaneously, training our neural networks on 1,000 devices in our training set yields a 10th quantile $R^2 >$ 0.995 on the test set. We achieve a similar R^2 when fitting 25 and 35 parameters after training on 4,000 and 16,000 devices, respectively (sample fits shown in **Supplementary Figures S6D-F**). In other words, within this range, we must roughly quadruple the size of the training set for every 10 additional fitting parameters to maintain the same level of accuracy.

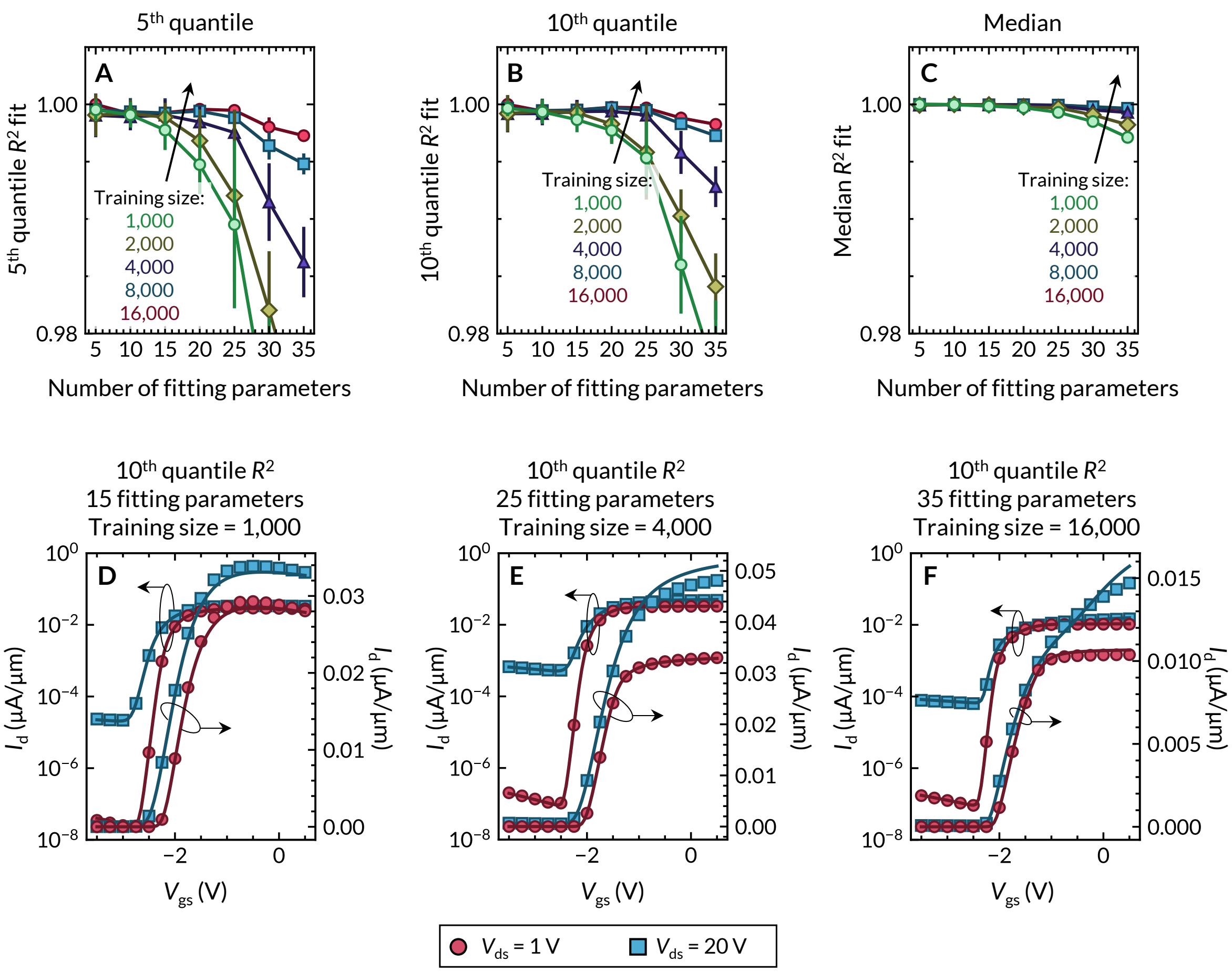

Supplementary Figure S6: Neural network performance *vs.* complexity of fitted models. Plots of (**A**) the 5th quantile R^2 fit, (**B**) the 10th quantile R^2 fit, and (**C**) the median R^2 fit plotted *vs.* the number of fitting parameters in the ASM-HEMT compact model. We show curves of the R^2 fit *vs.* number of fitting parameters as level curves for each training size tested. Markers and error bars show the means and standard deviations of the R^2 fit for five bootstrapped runs. We truncate the y-axis at $R^2 = 0.98$ to avoid overcrowding the plot. Panels (**D-F**) show the fits corresponding to the 10th quantile (worst 10%) R^2 from a bootstrapped model when fitting 15, 25, and 35 parameters simultaneously after training on data from 1,000, 4,000, and 16,000 devices, respectively. In all panels, we calculate R^2 using the procedure described in **Supplementary Section 4** across all V_{ds} values used; in panels d-f, we plot I_d-V_{gs} curves at only $V_{ds} = 1$ and 20 V to avoid cluttering the plots.

To understand how increasing the number of fitting parameters affects the extraction accuracy, we tabulate the median absolute error for each parameter across a sample bootstrap trial in **Supplementary Table S2**. For this comparison, we consider a fixed training set size of 16,000.

As expected, the error for each individual fitting parameters tends to increase with the number of parameters (i.e., moving left-to-right across a row in **Supplementary Table S2**). This occurs because the overall size of the parameter space increases, making fitting more difficult. Although the extraction error tends to increase as more fitting parameters are added, this upward trend is rarely monotonic; some parameter errors can decrease slightly as more fitting parameters are added, which could occur in part because of the stochasticity of the fitting process. Other parameters, especially ones with uniformly poor fits, have errors that are mostly independent of the number of fitting parameters, which could indicate weak correlation to the I_d-V_{gs} profile.

Supplementary Table S2: Median absolute errors for individual parameters when fitting HEMTs. The number in the top row (5, 10, … 35) indicates the number of device parameters fit simultaneously, and the tabulated value indicates the median absolute error for each fitting parameter in a test set of 1,000 devices. All parameter names and units are identical to those in the ASM-HEMT model. A superscripted asterisk (*) after the parameter name denotes that the parameter was sampled in logarithmic space (as in **Supplementary Table S1**); for these parameters, the median absolute error was calculated by first taking the base-10 logarithm of the true and predicted values. Parameter errors are listed as hyphens (-) when that parameter is held constant, i.e., when the parameter was set to a fixed value, rather than varying it.

**Number of fitting parameters**	**5**	**10**	**15**	**20**	**25**	**30**	**35**
**Parameter name**							
mexpaccs	0.961	0.984	0.955	0.885	0.954	0.908	0.956
thesat	6.70×10^{-4}	2.81×10^{-3}	5.02×10^{-2}	0.136	0.153	0.305	0.345
voff	1.19×10^{-4}	4.40×10^{-4}	2.16×10^{-3}	1.89×10^{-3}	5.98×10^{-3}	7.63×10^{-3}	5.88×10^{-3}
vsataccs	3.31×10^{4}	3.59×10^{4}	3.45×10^{4}	3.13×10^{4}	3.21×10^{4}	2.98×10^{4}	3.01×10^{4}
ub*	0.532	0.773	0.880	0.993	1.18	1.13	1.18
eta0	-	2.72×10^{-4}	5.78×10^{-4}	5.48×10^{-4}	6.98×10^{-4}	1.52×10^{-3}	1.13×10^{-3}
igddio	-	0.198	0.197	0.345	0.559	1.57	1.41
njgd	-	3.80	3.96	4.35	4.39	4.28	4.38
rdc	-	6.63×10^{-6}	2.65×10^{-5}	4.88×10^{-5}	1.21×10^{-4}	1.48×10^{-4}	1.71×10^{-4}
rnjgd	-	6.52×10^{-2}	5.85×10^{-2}	0.112	0.152	9.05×10^{-2}	7.05×10^{-2}
nfactor	-	-	5.33×10^{-3}	5.27×10^{-3}	1.11×10^{-2}	1.53×10^{-2}	1.14×10^{-2}
njgs	-	-	4.54	4.41	4.38	4.48	4.49
u0accs	-	-	1.30×10^{-2}	4.75×10^{-2}	5.01×10^{-2}	4.68×10^{-2}	4.84×10^{-2}
ua	-	-	4.52×10^{-9}	1.02×10^{-8}	2.99×10^{-8}	4.01×10^{-8}	4.00×10^{-8}
imin*	-	-	0.729	0.781	0.720	0.761	0.708
delta	-	-	-	0.236	0.498	0.597	0.568
gdsmin*	-	-	-	0.221	0.256	0.472	0.350
igsdio	-	-	-	1.86	1.77	1.89	1.97
lambda	-	-	-	4.46×10^{-4}	6.69×10^{-4}	7.48×10^{-4}	8.77×10^{-4}
ns0accs*	-	-	-	0.332	0.417	0.512	0.489
mexpaccd	-	-	-	-	0.954	0.913	0.873
rigsdio	-	-	-	-	2.30×10^{-8}	2.36×10^{-8}	2.27×10^{-8}
u0	-	-	-	-	1.66×10^{-2}	2.11×10^{-2}	1.96×10^{-2}
uted	-	-	-	-	2.94	3.07	3.30
ns0accd*	-	-	-	-	0.779	0.831	0.676
rigddio	-	-	-	-	-	6.84×10^{-9}	6.47×10^{-9}
rsc	-	-	-	-	-	1.70×10^{-4}	1.80×10^{-4}
rth0	-	-	-	-	-	6.35	6.49
utes	-	-	-	-	-	2.96	2.96
vdscale	-	-	-	-	-	0.114	0.135
cdscd	-	-	-	-	-	-	1.41×10^{-3}
rnjgs	-	-	-	-	-	-	2.47
u0accd	-	-	-	-	-	-	4.63×10^{-2}
ute	-	-	-	-	-	-	0.178
vsat	-	-	-	-	-	-	2.13×10^{4}

Supplementary References

1 Synopsys. Sentaurus Device Manual. (2017).

2 Liu, X., Choi, M. S., Hwang, E., Yoo, W. J. & Sun, J. Fermi Level Pinning Dependent 2D Semiconductor Devices: Challenges and Prospects. *Advanced Materials* **34**, 2108425, doi:10.1002/adma.202108425 (2022).

3 Bennett, R. K. A., Hoang, L., Cremers, C., Mannix, A. J. & Pop, E. Mobility and threshold voltage extraction in transistors with gate-voltage-dependent contact resistance. *npj 2D Materials and Applications* **9**, 13, doi:10.1038/s41699-024-00506-4 (2025).

4 Roy, S. & Bermel, P. Electronic and optical properties of ultra-thin 2D tungsten disulfide for photovoltaic applications. *Solar Energy Materials and Solar Cells* **174**, 370-379, doi:10.1016/j.solmat.2017.09.011 (2018).

5 Jo, S., Ubrig, N., Berger, H., Kuzmenko, A. B. & Morpurgo, A. F. Mono- and Bilayer WS2 Light-Emitting Transistors. *Nano Letters* **14**, 2019-2025, doi:10.1021/nl500171v (2014).

6 Falin, A. *et al.* Mechanical Properties of Atomically Thin Tungsten Dichalcogenides: WS_2, WSe_2, and WTe_2. *ACS Nano* **15**, 2600-2610, doi:10.1021/acsnano.0c07430 (2021).

7 Klinkert, C. *et al.* 2-D Materials for Ultrascaled Field-Effect Transistors: One Hundred Candidates under the Ab Initio Microscope. *ACS Nano* **14**, 8605-8615, doi:10.1021/acsnano.0c02983 (2020).

8 Evans, S. R., Deylgat, E., Chen, E. & Vandenberghe, W. G. Image-force barrier lowering of Schottky barriers in two-dimensional materials as a function of metal contact angle. *Physical Review Applied* **20**, 044003, doi:10.1103/PhysRevApplied.20.044003 (2023).

9 Hoang, L. *et al.* Understanding the Impact of Contact-Induced Strain on the Electrical Performance of Monolayer WS_2 Transistors. *Nano Letters* **24**, 12768-12774, doi:10.1021/acs.nanolett.4c02616 (2024).

10 He, Z., Yang, H. & Young Kim, N. Device simulation study of multilayer MoS_2 Schottky barrier field-effect transistors. *Nanotechnology* **36**, 035701, doi:10.1088/1361-6528/ad823e (2025).

11 Zhao, Y. *et al.* Electrical spectroscopy of defect states and their hybridization in monolayer MoS_2. *Nature Communications* **14**, 44, doi:10.1038/s41467-022-35651-1 (2023).

12 Jana, K. *et al.* in *2024 International Conference on Simulation of Semiconductor Processes and Devices (SISPAD).* 01-04.

13 Wang, X. & Dodabalapur, A. Carrier Velocity in Amorphous Metal–Oxide–Semiconductor Transistors. *IEEE Transactions on Electron Devices* **68**, 125-131, doi:10.1109/TED.2020.3037890 (2021).

14 Liu, Y., Zeng, J., Zhu, Z., Dong, X. & Deng, W. A physics-based compact model for MoS_2 field-effect transistors considering the band-tail effect and contact resistance. *Japanese Journal of Applied Physics* **59**, 104004, doi:10.35848/1347-4065/abb7e1 (2020).

15 Chiquito, A. J. *et al.* Back-to-back Schottky diodes: the generalization of the diode theory in analysis and extraction of electrical parameters of nanodevices. *Journal of Physics: Condensed Matter* **24**, 225303, doi:10.1088/0953-8984/24/22/225303 (2012).

16 Nasr, J. R., Schulman, D. S., Sebastian, A., Horn, M. W. & Das, S. Mobility Deception in Nanoscale Transistors: An Untold Contact Story. *Advanced Materials* **31**, 1806020, doi:10.1002/adma.201806020 (2019).

17 Bennett, R. K. A. *GitHub Repository.* Available online: https://github.com/RKABennett/deep-learning-for-transistor-parameter-extraction (2025).

18 Dasgupta, A., Ghosh, S., Chauhan, Y. S. & Khandelwal, S. in *2015 IEEE International Conference on Electron Devices and Solid-State Circuits (EDSSC).* 495-498.

19 Martinez, R. P. *et al.* Compact Model Parameter Extraction via Derivative-Free Optimization. *IEEE Access* **12**, 123224-123235, doi:10.1109/ACCESS.2024.3453198 (2024).

20 Köppen, M. in *5th online world conference on soft computing in industrial applications (WSC5).* 4-8.
